# Exosome-mediated mRNA delivery *in vivo* is safe and can be used to induce SARS-CoV-2 immunity

**DOI:** 10.1016/j.jbc.2021.101266

**Published:** 2021-10-01

**Authors:** Shang Jui Tsai, Nadia A. Atai, Mafalda Cacciottolo, Justin Nice, Arjang Salehi, Chenxu Guo, Alanna Sedgwick, Saravana Kanagavelu, Stephen J. Gould

**Affiliations:** 1 Department of Biological Chemistry, The Johns Hopkins University School of Medicine, Baltimore, Maryland, USA; 2 Capricor Therapeutics, Inc, Beverly Hills, California, USA

**Keywords:** COVID19, spike, nucleocapsid, exosome, mRNA, cationic lipid, lipofection, antibody, T-cell, extracellular vesicles, ACE2, angiotensin-converting enzyme II, BCA, bicinchoninic acid, BLI, bioluminescent imaging, CDM, chemically defined media, CTCS, clarified tissue culture supernatant, CVS, concentrated vesicle suspension, DOPE, dioleoyl phosphatidylethanolamine, DOTAP, dioleoyl-3-trimethylammonium propane, DTZ, diphenylterazine, ER, endoplasmic reticulum, EV, extracellular vesicle, HUVEC, human umbilical vein endothelial cell, LNP, lipid nanoparticle, NTA, nanoparticle tracking analysis, ORF, open reading frame, pfPBS, particle-free PBS, SARS-CoV-2, severe acute respiratory syndrome coronavirus 2, SEC, size-exclusion chromatography

## Abstract

Functional delivery of mRNA has high clinical potential. Previous studies established that mRNAs can be delivered to cells *in vitro* and *in vivo via* RNA-loaded lipid nanoparticles (LNPs). Here we describe an alternative approach using exosomes, the only biologically normal nanovesicle. In contrast to LNPs, which elicited pronounced cellular toxicity, exosomes had no adverse effects *in vitro* or *in vivo* at any dose tested. Moreover, mRNA-loaded exosomes were characterized by efficient mRNA encapsulation (∼90%), high mRNA content, consistent size, and a polydispersity index under 0.2. Using an mRNA encoding the red light-emitting luciferase Antares2, we observed that mRNA-loaded exosomes were superior to mRNA-loaded LNPs at delivering functional mRNA into human cells *in vitro*. Injection of Antares2 mRNA-loaded exosomes also led to strong light emission following injection into the vitreous fluid of the eye or into the tissue of skeletal muscle in mice. Furthermore, we show that repeated injection of Antares2 mRNA-loaded exosomes drove sustained luciferase expression across six injections spanning at least 10 weeks, without evidence of signal attenuation or adverse injection site responses. Consistent with these findings, we observed that exosomes loaded with mRNAs encoding immunogenic forms of the SARS-CoV-2 Spike and Nucleocapsid proteins induced long-lasting cellular and humoral responses to both. Taken together, these results demonstrate that exosomes can be used to deliver functional mRNA to and into cells *in vivo*.

The functional delivery of RNA molecules is an emerging approach to vaccination and therapy (1, 2). Although naked mRNA delivery has been reported (3, 4, 5), its efficacy is limited by high rates of plasma RNA degradation, poor cellular entry, and nucleic-acid-induced inflammatory responses (6, 7, 8, 9). Formulation of RNAs into lipid nanoparticles (LNPs) has been developed as a way to overcome these limitations (10), but LNPs and other synthetic nanoparticles are foreign entities, and LNP-associated side effects range from mild to severe (1, 11). In contrast to LNPs and other synthetic RNA delivery particles, exosomes are small secreted vesicles (∼30–200 nm in dia.) that are produced by all cells, are present at high concentrations in interstitial and other body fluids, and have been present throughout the life of every person and the evolution of every animal (12). In fact, exosomes are the only biologically normal (bionormal) nanovesicle in existence.

As first reported by Trams *et al*. (13), mammalian cells normally secrete two types of extracellular vesicle (EV), smaller vesicles of ∼30–200 nm dia. that are commonly referred to as exosomes and larger vesicles (typically >300 nm dia.) that are commonly referred to as microvesicles (12, 14). Exosomes are highly enriched in specific subsets of proteins, RNAs, and lipids, providing strong evidence that they are generated by an active sorting and vesicle biogenesis pathway (12, 15, 16, 17). In contrast, microvesicles have a molecular composition that is closer to that of the cell, and there is little if any evidence for a selective biogenesis pathway for this class of larger EVs. As for how cells make exosomes, there is compelling empirical evidence that their structural and composition heterogeneity is generated *via* a single, shared pathway of vesicle budding that occurs across a spectrum of plasma and endosome membranes (12, 18). We therefore use the term exosome to refer to all exosome-sized vesicles that are secreted by the cell (12).

Once released into the extracellular milieu, exosomes can transmit signals and molecules to other cells (19). Consistent with their natural ability to transfer RNAs between distinct cells, several groups have demonstrated that these bionormal nanovesicles can be loaded with synthetic small RNAs (20, 21, 22). Even more, we and others have demonstrated that RNA-loaded exosomes can be used to deliver anticancer RNAs to and into tumors and tumor cells, inhibiting the expression of the target mRNAs, suppressing tumor growth and extending the life span of tumor-bearing animals (20, 21).

The ability of exosomes to deliver functional RNAs is particularly notable given the success of mRNA-based vaccines in the fight against COVID-19/severe acute respiratory syndrome coronavirus 2 (SARS-CoV-2) (23, 24, 25, 26, 27, 28, 29, 30, 31, 32, 33, 34, 35, 36, 37, 38). Nearly all of these vaccines are designed to elicit immunity through the expression of SARS-CoV-2 Spike protein, which mediates the binding of virus particles to receptors on the host cell surface (primarily angiotensin-converting enzyme II [ACE2] (23, 39, 40) but also neuropilin-1 (41, 42)) and catalyzes the fusion of virus and cell membranes (39, 43). These Spike-only vaccines have proved to be remarkably effective at reducing the incidence of SARS-CoV-2-associated morbidity and mortality. However, there is increasing evidence that their protective effect is manifest primarily in the respiratory tract, the proximal site of infection, with lower efficacy against SARS-CoV-2 infection of, and damage to, distal sites such as the brain (44).

Recent evidence suggests that vaccination with both the Nucleocapsid and Spike proteins results in better protection against the proximal and distal consequences of SARS-CoV-2 infection (44, 45, 46, 47). As a result, incorporation of both antigens in a vaccine strategy is likely to elicit stronger and broader protection against SARS-CoV-2 disease. Another argument in favor of a multiplexed Spike and Nucleocapsid vaccine is that Nucleocapsid is far more conserved between different strains of SARS-CoV-2 and therefore more likely to offer similar protection against different strains of this virus. This may be particularly important given the rise of SARS-CoV-2 variants such as delta, which has an enhanced ability to infect and cause disease in vaccinated individuals (48, 49). The rationale for inclusion of Nucleocapsid in a multiplexed vaccine is also supported by the observations that Nucleocapsid is highly expressed in infected cells, is a major target of the immune response in COVID-19 patients, is released from infected cells and activates complement as a free soluble protein (50), and has been used to elicit strong immunity against the SARS Nucleocapsid protein (51). In addition, Nucleocapsid-targeted vaccines have shown some ability to protect against COVID-19 disease (44, 52).

While LNPs have proved effective at delivering mRNA-based Spike-expressing vaccines, there is increasing evidence of LNP-associated adverse effects (10, 53, 54). Taken together, these considerations warrant an investigation of exosomes as a delivery vehicle for mRNAs encoding both Spike and Nucleocapsid antigens. Here we report a procedure for generating mRNA-loaded human exosomes, an analysis of their efficacy in functional mRNA delivery, their utility for driving mRNA-templated protein expression *in vivo* in eyes and muscle, and the ability of a multiplexed, mRNA-loaded exosome formulation to elicit humoral and cellular immunity to SARS-CoV-2 Spike and Nucleocapsid proteins.

## Results

### Exosome production, purification, and characterization

**Figure 1:**
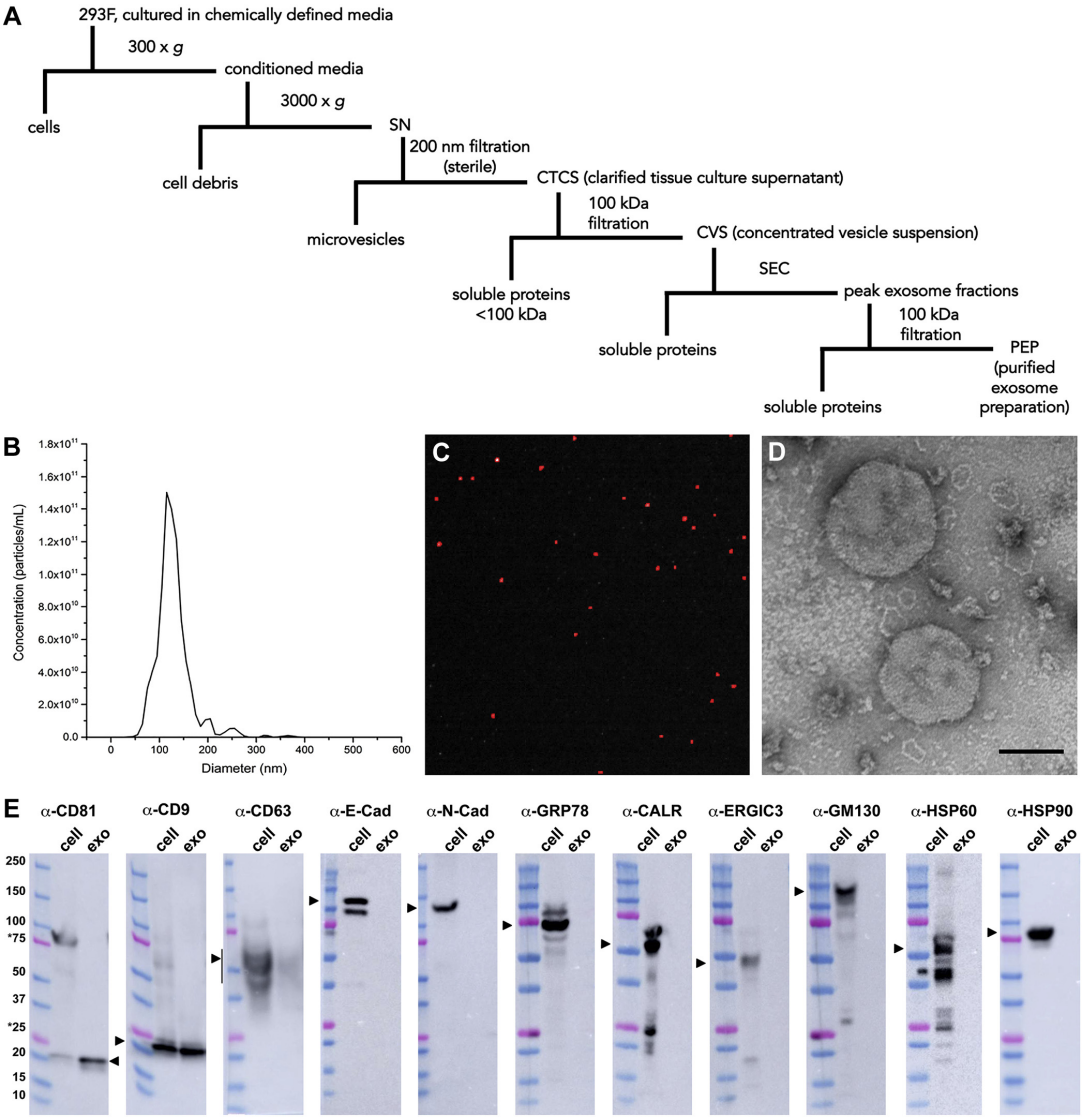
**Exosome purification and characterization.***A*, schematic describing exosome purification procedure. *B*, size distribution plot of purified exosomes, as determined by NTA. *C*, immunofluorescence image of purified exosomes, labeled with an Alexa Fluor 647-conjugated anti-CD63 monoclonal antibody, captured using the Particle Metrix PMX-220 ZetaView camera. *D*, electron micrograph of negative-stained, purified exosomes. Bar, 100 nm. *E*, immunoblot analysis of equal proportions of 293F cell and exosome lysates using antibodies specific for the exosomal markers CD81, CD9, CD63, E-cadherin (E-cad), N-cadherin (N-cad), GRP78/BiP, calreticulin (CALR), ERGIC-3, GM130, HSP60, and HSP90. The amount and ratio of cell and exosome lysates were selected empirically to show the different enrichment of the CD81, CD9, and CD63 proteins, were kept constant in all immunoblots, by proportion equaled a 10-fold overloading of exosomes relative to cells, and by amount of protein equaled 15 μg cell lysate protein/lane and 0.15 μg exosome lysate protein/lane.

HEK293 cells are the only extensively studied, immortalized human cell line that was not derived from a human cancer (55). 293F cells were generated as a spontaneously arising single cell clone of HEK293 cells that has the properties of rapid growth, the ability to grow in suspension in chemically defined media (CDM), and facile use for recombinant protein production (56). These traits indicate that 293F cell cultures are a good starting material for the purification of human exosomes that are free of animal products. To confirm that 293F-derived exosomes could be produced by a scalable approach, 293F cells were grown in CDM at a starting density of ∼1–1.5 × 10^6^ cells/ml and grown for 3 days, with shaking (Fig. 1*A*). At the conclusion of this incubation, cells and large cell debris were removed by low-speed centrifugation to generate a conditioned media supernatant. This was subjected to gravity filtration across an ∼200 nm pore size diameter sterile filter, removing microvesicles and other large EVs from the sample. The flow-through represents a clarified tissue culture supernatant (CTCS) that had a volume roughly equal to the culture volume, a protein concentration of ∼1 mg/ml, and an exosome concentration of ∼1 × 10^10^ exosomes/ml. Next, exosomes were concentrated from the CTCS by ∼100-fold by filtration across a 100 kDa pore size membrane to produce a concentrated vesicle suspension (CVS). The CVS had 1/100th the volume, 1/20th the protein, yet retained more than ½ the exosomes present in the CTCS. Exosomes were purified from the CVS by size-exclusion chromatography (SEC), with exosomes eluting immediately following the void volume yet prior to most (∼95%) soluble proteins. Peak exosome fractions from the SEC column were then pooled and further concentrated by filtration, with each 1 L of 293F culture yielding ∼5 × 10^12^ exosomes.

The exosomes generated by this procedure were interrogated for vesicle size, identity, specificity, and overall molecular composition. Nanoparticle tracking analysis (NTA) revealed that these exosomes have an average diameter of ∼120 nm ± 50 nm (s.e.m.) (Fig. 1*B*). When preincubated with fluorescently labeled anti-CD63 antibodies and then re-examined by fluorescence-triggered NTA, these exosomes were found to carry the exosome marker protein CD63 epitope on their outer surface (Fig. 1*C*). Furthermore, negative-stain electron microscopy confirmed that these exosomes fell within the size range calculated by NTA (Fig. 1*D*). Immunoblot analysis of cell and exosome fractions confirmed the enrichment of classic exosome marker proteins CD81, CD9, and CD63 in the exosome preparation (Fig. 1*E*). These same exosome fractions lacked detectable levels of protein markers of the plasma membrane and microvesicles (E-cadherin and N-cadherin), endoplasmic reticulum (ER) and nuclear envelope (GRP78/BiP and calreticulin), ER-Golgi intermediate compartment (ERGIC3), Golgi (GM130), mitochondria (HSP60), or exomeres (HSP90) (57, 58). As for their overall macromolecular content, these exosomes had a protein concentration of ∼1 mg/10^12^ exosomes, a lipid concentration of ∼0.1 mg/10^12^ exosomes, an RNA concentration of ∼0.02 mg/10^12^ exosomes, and only trace amounts of DNA, most of which were under 100 basepairs in length (Fig. S1).

### 293F-derived exosomes are safe and nontoxic

**Figure 2:**
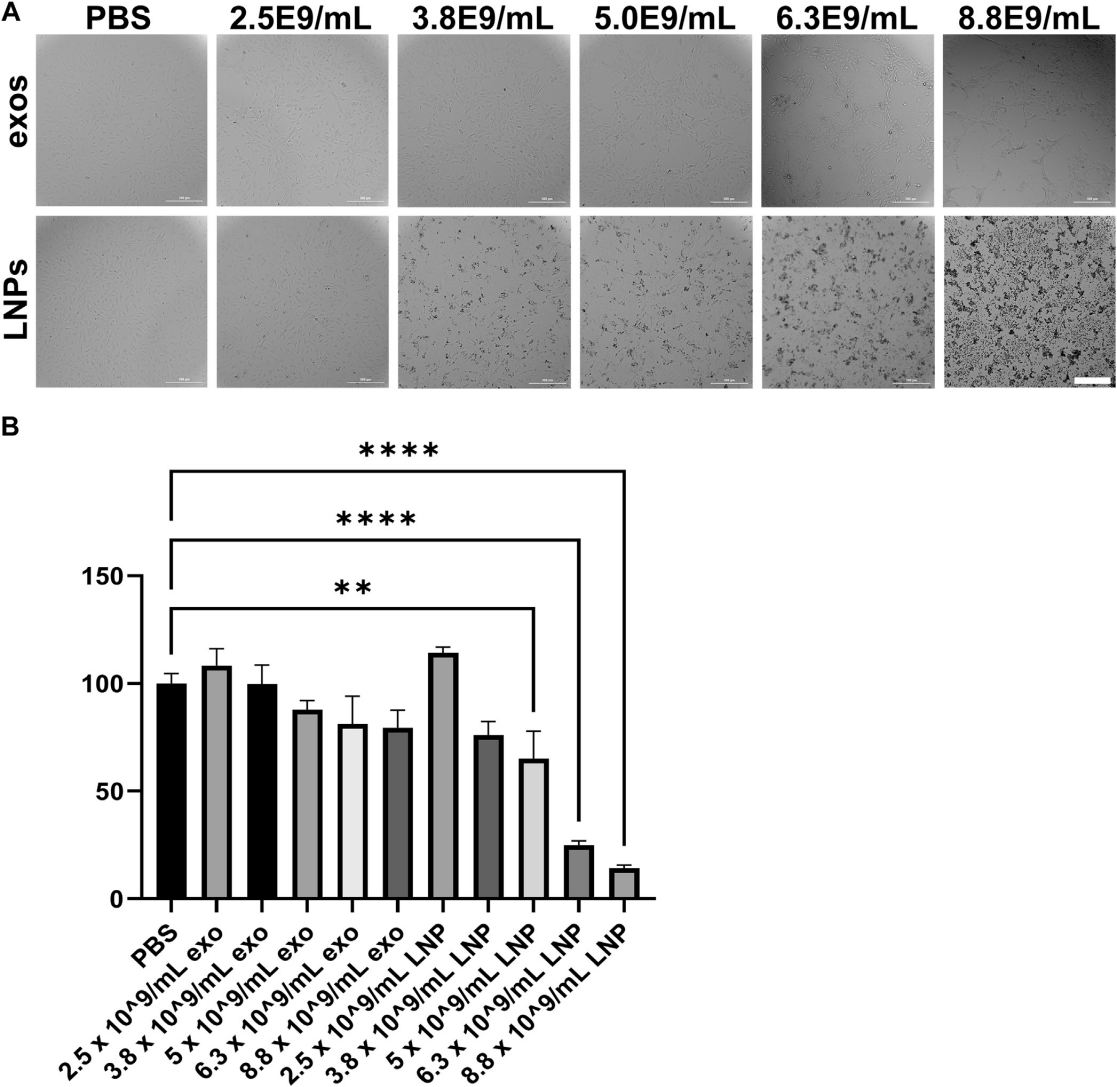
**Exosomes are well tolerated, LNPs are not.***A*, light micrographs of HUVECs following a 24 h-long incubation with PBS or with purified exosomes or LNPs at matched concentrations of particles, as determined by NTA. Bar, 0.3 mm. *B*, bar graph of MTT measurements of HUVECs following a 24-h-long incubation with PBS or with purified exosomes or LNPs at matched concentrations of particles, normalized to PBS control (100). Bar height equals the average, hatched line represents the standard error of the mean, and asterisks denote differences that were statistically significant (n = 8, one-way ANOVA; ∗∗ <0.01, ∗∗∗ <0.001, ∗∗∗∗ <0.0001).

As the only bionormal nanovesicle, exosomes are predicted to be safer and less toxic than synthetic, chemically synthesized nanoparticles such as LNPs. To test this hypothesis, we exposed primary human umbilical vein endothelial cells (HUVECs) to equal numbers of (i) 293F-derived exosomes and (ii) a prototypical LNP comprised of dioleoyl-3-trimethylammonium propane (DOTAP) and dioleoyl phosphatidylethanolamine (DOPE) (59). Following an overnight incubation, these particle-treated HUVECs (and PBS-treated control cells) were examined by light microscopy (Fig. 2*A*), revealing that LNP-treated cells displayed a pronounced, dose-dependent cytopathic effect, whereas exosome-treated cells did not. Particle-treated HUVECs were also interrogated by the 3-(4,5-dimethylthiazol-2-yl)-2,5-diphenyltetrazolium bromide (MTT) assay, which measures mitochondrial oxidative capacity (60). These experiments revealed that LNPs exposure elicited a dose-dependent inhibition of MTT activity, whereas exosomes did not (Fig. 2*B*).

**Figure 3:**
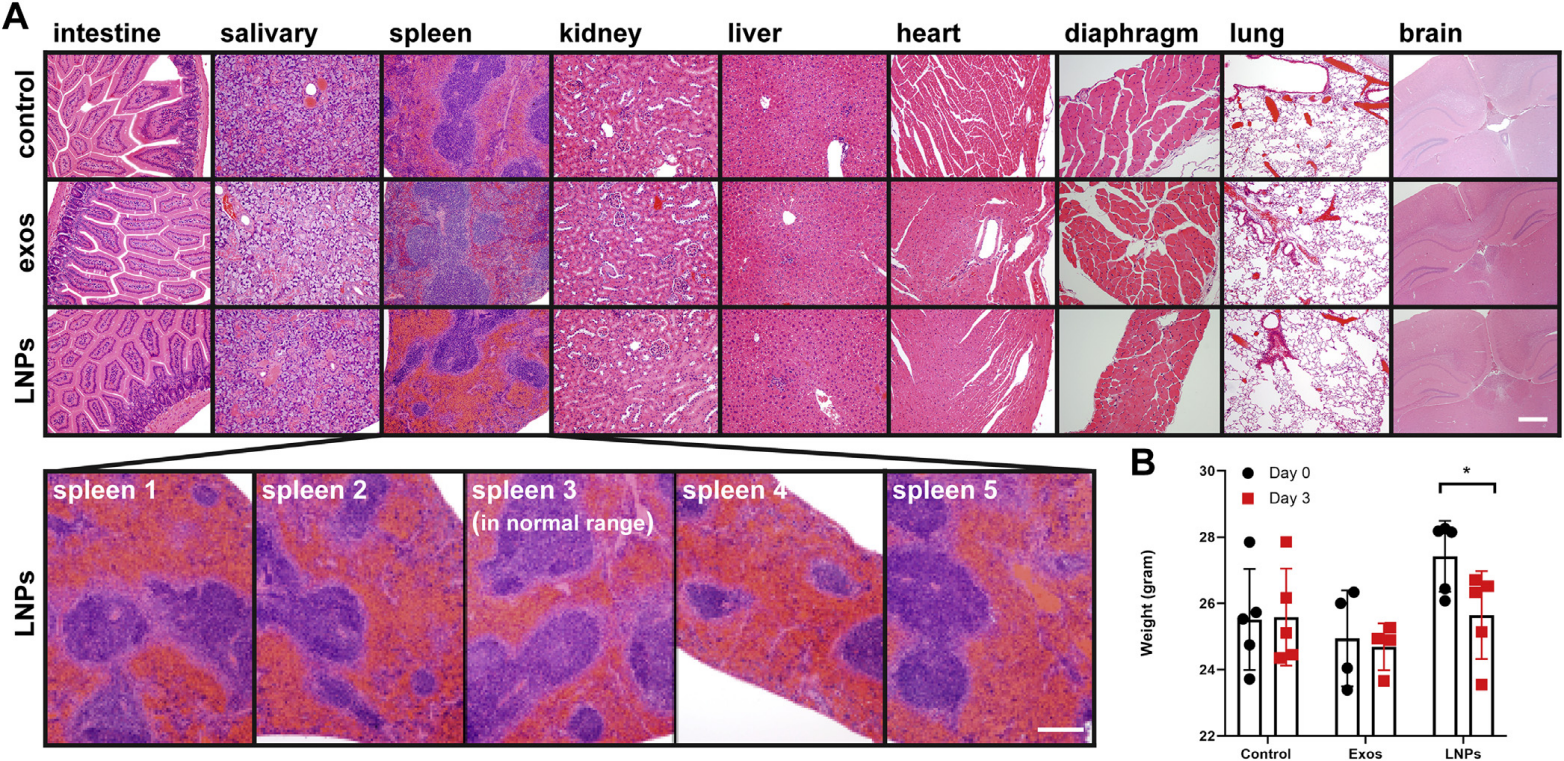
**Effect of exosome and LNP injections on organ histology and body mass**. *A*, hematoxylin and eosin staining of tissue sections from BALB/c mice that had been injected 3 days earlier with 0.050 ml of PBS, exosomes (1.5 × 10^10^), or LNPs (1.5 × 10^10^). Note the hypertrophy of spleen red pulp in 4/5 animals injected with LNPs. Bar, 0.2 mm. *B*, bar graph displaying body mass measurements prior to and at 3 days after injection.

We next tested the effect of exosomes and LNPs on live animals. Mice were injected intramuscularly (i.m.) with 0.05 ml of (i) PBS, (ii) PBS containing 1.5 × 10^10^ exosomes, or (iii) PBS containing 1.5 × 10^10^ LNPs (five animals each). Three days later, the mice were examined visually, weighed, and sacrificed for tissue histological analysis by an independent pathology service (University of Washington). The pathologists' report concluded that all exosome-injected animals fell within the normal range. LNP-injected animals were also normal in nearly all respects, with the exception that 4/5 LNP-injected animals displayed an increase in spleen red pulp (Fig. 3*A*), perhaps indicative of mild splenic congestion. Visual inspection of the animals revealed that there were no injection site reactions or any other adverse signs of distress. In fact, the only clear difference in the animals was that the LNP-injected animals exhibited a slight (∼5%) decline in body mass (Fig. 3*B*).

### Creation of Antares2 mRNA-loaded exosomes

To determine whether exosomes could be loaded with exogenous, *in vitro* synthesized mRNAs, we first designed and synthesized a test mRNA that encodes an easily assayed proxy marker of functional mRNA delivery, Antares2 (61). Antares2 is a CyOFP1-teLuc-CyOFP1 fusion protein comprised of two copies of CyOFP1 (an orange-red emitting, teal-light-excited fluorescent protein (62)), separated by the teal-light-emitting luciferase teLuc (61). Oxidation of the luciferin diphenylterazine (DTZ) by Antares2 leads to orange-red light emission, rather than the blue light emitted by teLuc alone, due to bioluminescence resonance energy transfer (BRET) in which the energy of DTZ oxidation is transferred to the fluorophores of the CyOFP1 moieties. This results in a pronounced shift in the wavelength of emitted light, which is critical to the detection of functional mRNA delivery *in vivo*, due to the inverse proportionality between the wavelength of light and its absorbance by tissue (63).

**Figure 4:**
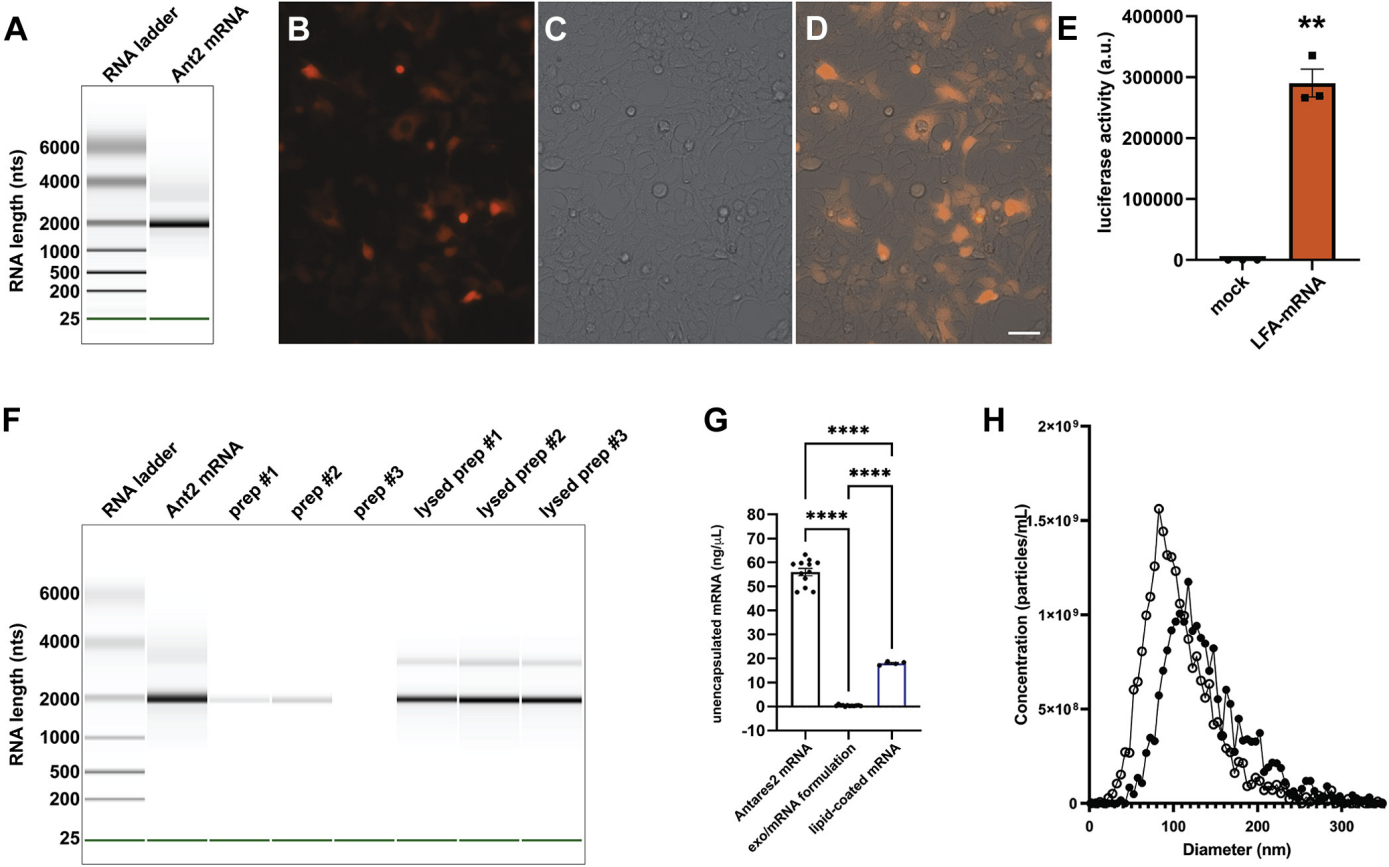
**Characterization of Antares2 mRNA and mRNA-loaded exosomes.***A*, gel-like image of *in vitro* synthesized Antares2 mRNA interrogated using an RNA chip on an Agilent Bioanalyzer. *B–D*, fluorescence and brightfield micrographs of HEK293 cells 2 days after transfection with Antares2 mRNA. Cells were grown on sterile, poly-L-lysine-coated coverglasses, fixed, mounted, and imaged using an EVOS M7000 microscope system. Bar, 50 μm. *E*, bar graph of luciferase activity of HEK293 cells measured 2 days after transfection with Antares2 mRNA. Cells were grown in 96 well plates, DTZ solution was added, and light emission measured using a plate reader. Bar height represents average light emission, hatched line represents standard error of the mean, and asterisks represent Student's *t* test, with ∗∗ denoting *p* < 0.005. *F* and *G*, efficient encapsulation of mRNA by lipid-mediated exosome loading. *F*, gel-like image of RNA markers and samples separated on an RNA chip in an Agilent Bioanalyzer. Data is presented, left to right, for Antares2 mRNA, three separate mRNA-loaded exosome preparations, and the RNA extracted from these same preparations. *G*, bar graph of Ribogreen fluorescence-based measurement of free accessible RNA concentrations, measured using a plate reader relative to RNA standards of known concentration, for matched samples of Antares2 mRNA, Antares2 mRNA-loaded exosomes, and Antares2 mRNA coated with cationic lipids (n ≥ 6; bar height represents the average, hatched lines represent standard error of the mean, and asterisks denote statistical significance calculated by one-way ANOVA, with ∗∗∗∗ <0.0001). *H*, size distribution profile of (*open circles*) purified exosomes and (*closed circles*) exosomes that had been loaded with Antares2 mRNA, as determined by NTA.

The Antares2 open reading frame (ORF) is 1882 nucleotides (nts)-long, and with polyA tail and untranslated regions, was expected to be ∼2000 nts in length. Examination of the *in vitro* synthesized Antares2 mRNA using a bioanalyzer (Agilent) confirmed that the Antares2 mRNA sample ran as a single band of ∼2000 nts (Fig. 4*A*). To assess its functionality, the Antares2 mRNA was transfected into 293F cells, which interrogated the next day by fluorescence microscopy and luciferase assay. Upon excitation with green light, a significant proportion of the transfected HEK293 cells emitted orange-red light, as expected for Antares2 (Fig. 4, *B–D*). Furthermore, when the same cell population was exposed to a cell-permeable luciferin substrate, DTZ, the transfected cell population displayed 3000-fold more light emission (n = 3; Student's *t* test *p* = 0.001) than the untransfected 293F cells (Fig. 4*E*) (the background light emission is likely due to a low rate of spontaneous, uncatalyzed oxidation of DTZ).

To load the *in vitro* synthesized Antares2 mRNA into exosomes, we first mixed it with cationic lipids (20) to generate lipid-coated mRNAs (64), and then loaded the lipid-coated mRNA into exosomes by mixing-induced partitioning. Both processes are driven by the attractive force of water, resulting in the encapsulation of lipid-coated mRNAs into exosomes and exosome membranes. To determine the efficiency of this process, the products of three independent mRNA-loading reactions were examined using an RNA-chip on an Agilent bioanalyzer (Fig. 4*F*). These experiments indicated that >90% (92.5%, n = 3) of the input mRNA was encapsulated within exosomes, leaving <10% available for detection on the bioanalyzer. Furthermore, when these mRNA-loaded exosomes were subject to lysis and RNA extraction, we recovered ∼80% of the input mRNA, with no evidence of mRNA degradation during either the loading or lysis procedures (Fig. 4*F*). RNA encapsulation was also measured using the RiboGreen RNA detection assay, in which intercalation of the RiboGreen dye into mRNA results in RiboGreen fluorescence (65). Application of the RiboGreen assay to purified Antares2 mRNA, mRNA-loaded exosomes, and lipid-coated mRNAs revealed that less than 1% of input mRNA was accessible to the RiboGreen dye (Fig. 4*G*). Furthermore, the amount of free, unincorporated mRNA was 30-fold lower in the mRNA-loaded exosomes than in the intermediate lipid-coated mRNA solution.

To better understand how this process may affect the exosome, NTA analysis was performed on exosomes prior to and after mRNA loading (Fig. 4*H*). These experiments showed that the mRNA-loading procedure caused only minor shifts in vesicle concentration (20% reduction), size (20% increase), and polydispersity index (20% reduction) (Table 1). As expected, electron microscopic analysis of exosomes, lipid-mRNA complexes, and mRNA-loaded exosomes confirmed that each step in the creation of mRNA-loaded exosomes resulted in a physical transformation of the reactants, with the mRNA-loaded exosomes having a distinct morphology from both the lipid-coated mRNAs and the purified exosomes used in the loading reaction (Fig. S2).

**Table 1:** Effect of mRNA loading process on exosome concentration, size, and PDI, as determined by NTA

Sample	Concentration	Diameter	PDI
Exosomes prior to loading	3.0 × 10^10^	120 nm ± 50	0.17 ± 0.02
Exosomes after loading with mRNA	2.4 × 10^10^	140 nm ± 50	0.14 ± 0.01

### mRNA-loaded exosomes drive higher mRNA expression than mRNA-loaded LNPs

**Figure 5:**
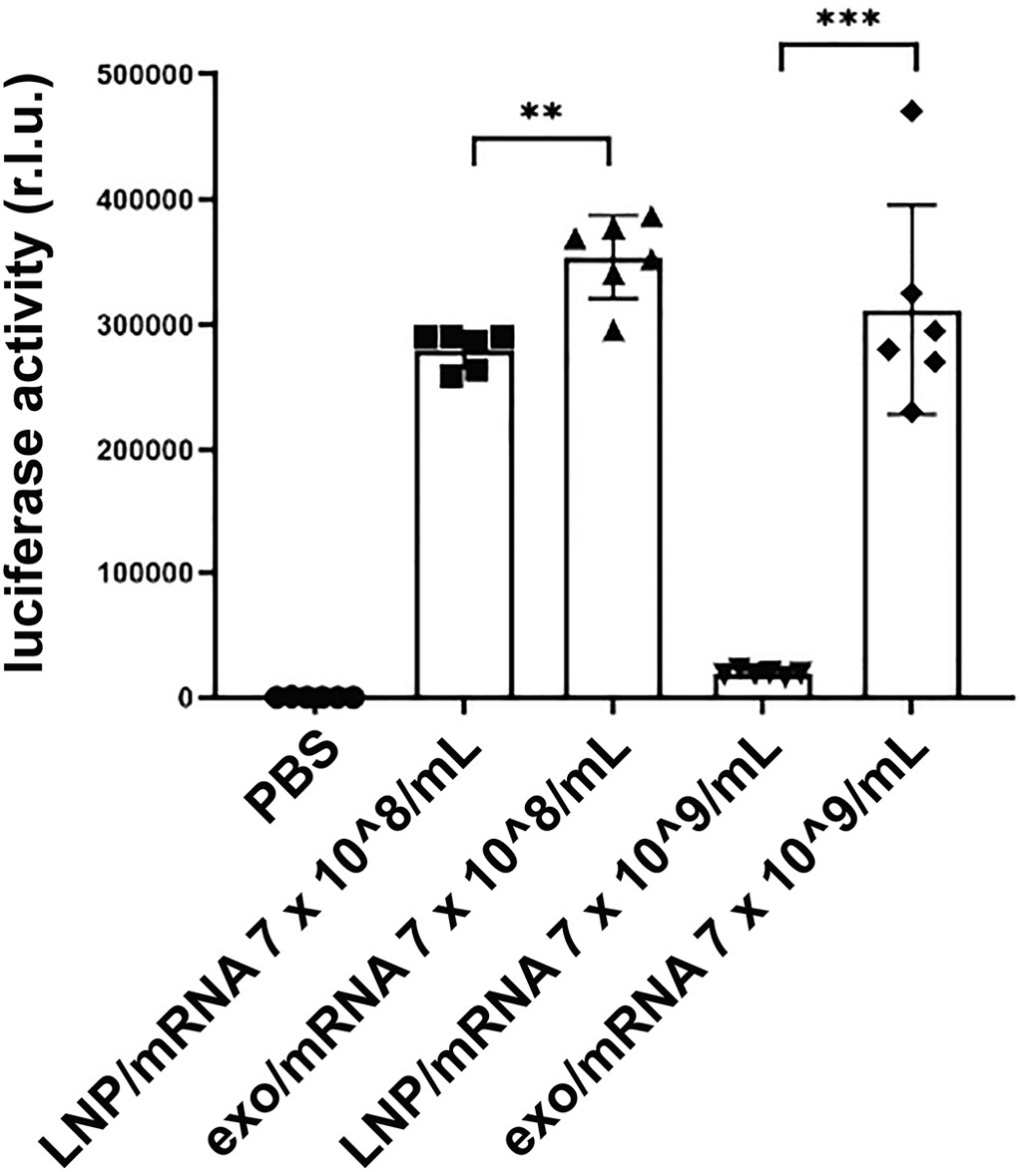
**mRNA-loaded exosomes deliver functional Antares2 mRNA to human cells.** Bar graph of luciferase activities exhibited by 293F cells treated for 1 day with PBS, Antares2 mRNA-loaded exosomes, or Antares2 mRNA-loaded LNPs, each of which was added at two different concentrations (bar height equal the average, error bars represent standard error of the mean, and asterisks denote significance as determined by one-way ANOVA (n = 6; ∗∗ <0.01, ∗∗∗ <0.001)).

We next tested whether exosomes loaded with the Antares2 mRNA could deliver functional Antares2 mRNA into human cells, and whether it could do so at an efficiency similar to that of LNPs. Specifically, we loaded Antares2 mRNA into purified exosomes and also into LNPs, added the resulting formulations to cultures of human cells, grew the cells overnight to allow for mRNA uptake and expression, and then assayed the cells for Antares2 luciferase activity (Fig. 5). These experiments established that mRNA-loaded exosomes were able to induce high levels of Antares2 enzyme expression in the treated cells. In fact, the level of Antares2 expression was higher in cells exposed to mRNA-loaded exosomes than in cells exposed to mRNA-loaded LNPs. The superiority of Antares2 mRNA expression in the exosome-treated samples was relatively modest at low dose (30% increase; n = 6, *p* = 0.0016) but dramatic at high dose (16-fold increase; n = 6, *p* = 0.0004).

### Exosome-mediated mRNA expression in the eye and muscle

**Figure 6:**
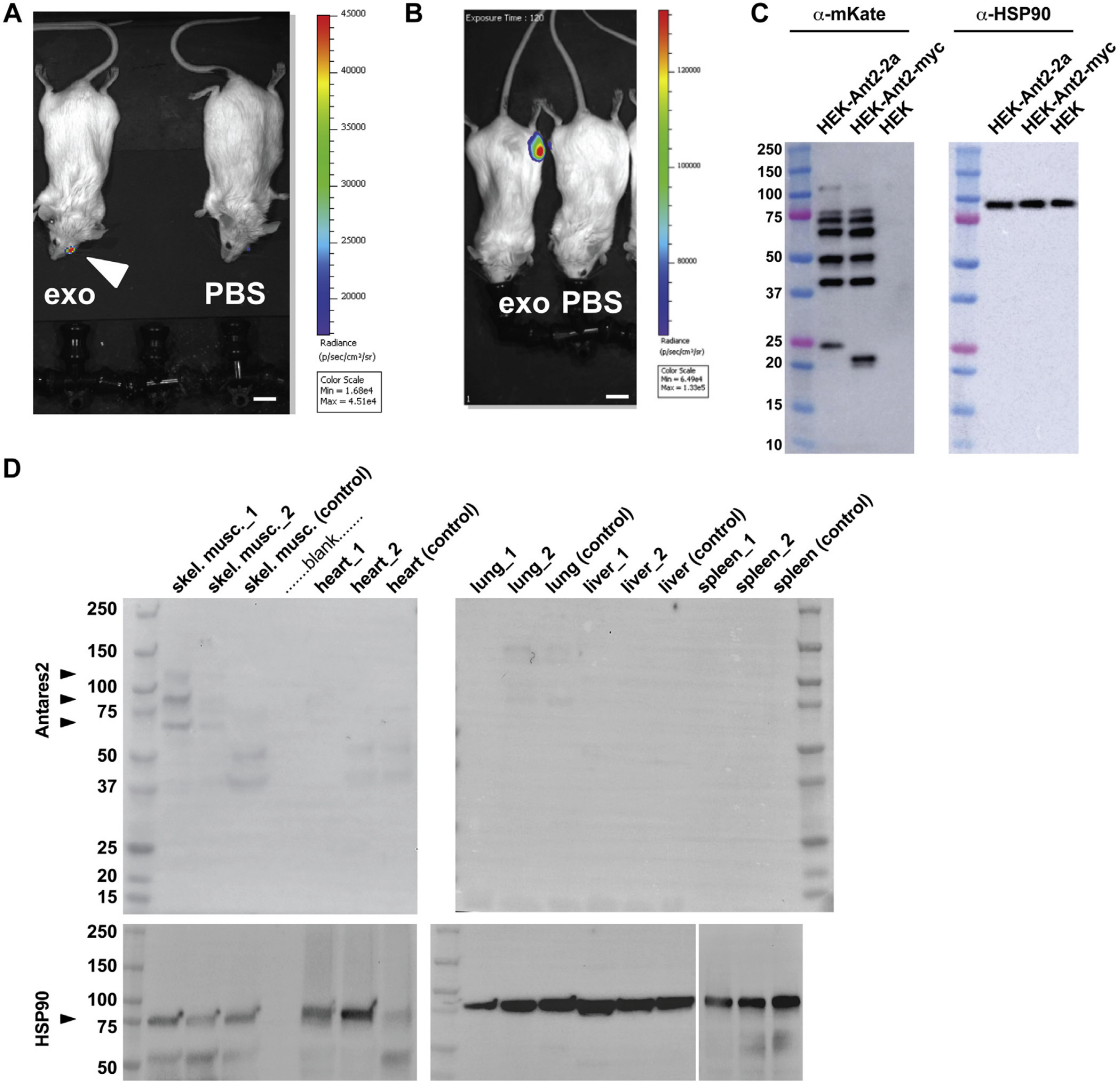
**Antares2 expression in vivo following injection of Antares2 mRNA-loaded exosomes.***A*, combined light and bioluminescence images showing DTZ-triggered, Antares2-catalyzed light emission from the eye of mice that had been injected intravitreally with exosome/mRNA formulation and then exposed to topical DTZ solution 36 h later. Light emission, measured in radiance (photons/s/area (cm^2^)/steradian), is reflected in the color, with red denoting the areas of highest light emission and blue denoting the areas of lower but still significant light emission. Similar results were observed in a second animal that was subjected to the same treatment. Bar, 1 cm. *B*, combined light and bioluminescence images showing DTZ-triggered, Antares2-catalyzed light emission from the muscle of mice that had been injected i.m. in the left thigh with exosome/mRNA formulation and reinjected in both legs i.m. with DTZ solution 48 h later. This experiment was performed in a single animal. Bar, 1 cm. *C*, immunoblot of cell lysates prepared from HEK293 cells (HEK) and from cells that had been transfected 2 days earlier with plasmids designed to express Antares2-2a and Antares2-myc, using anti-mKate antibodies to detect the products of Antares2 expression and anti-HSP90 antibodies to confirm the presence of HEK293 proteins in each sample. *D*, immunoblot of tissue protein extracts collected 2 days after mice had been injected i.m. with either mRNA-loaded exosomes (animals 1 and 2) or PBS (control). Tissue protein samples were probed with (*upper blots*) antibodies specific for mKate to detect Antares2 and with (*lower blots*) antibodies specific for HSP90. *Arrowheads* denote position of Antares2 proteins detected by the anti-mKate antibodies in skeletal muscle of treated animals.

Taken together, the superiority of exosomes at delivering functional mRNAs and the cytopathic effects of LNPs led us to restrict our *in vivo* studies to mRNA-loaded exosomes. Furthermore, the ability to detect exosome-mediated mRNA delivery *via* Antares2 expression led us to test for *in vivo* mRNA in the eye, where the Antares2 substrate DTZ could be applied topically to the outer surface. The vitreous of the eye is an isolated biofluid that exchanges relatively slowly with other body fluids, and thus, intravitreal injection of mRNA-loaded exosomes should lead to Antares2 expression in cells of the eye. Toward this end, anesthetized mice were injected intravitreally with either 2 μL Antares2 mRNA-loaded exosomes (∼6 × 10^7^ total particles) or 2 uL of PBS and then returned to their cages. Thirty-six hours later, the mice were reanesthetized and placed in a black box imaging system. A drop (∼0.04 ml) of 0.01 mM DTZ (in PBS) was applied to the surface of the injected eye and the animals were examined by bioluminescent imaging (BLI). This experiment revealed that there was strong DTZ-induced light emission from the injected eye, which reached a peak radiance of ∼45,000 p/s/cm^2^/sr (Fig. 6*A*). As mouse eyes do not normally emit light, these results represent strong evidence that mRNA-loaded exosomes can drive functional protein expression and enzyme activity in cells of the mammalian eye.

To determine whether mRNA-loaded exosomes could drive Antares2 expression in other tissues, we injected 0.05 ml of Antares2 mRNA-loaded exosomes into thigh muscle. Two days later, the mice were again anesthetized, placed in a black box imaging system, and then injected with 0.05 ml of 0.01 mM DTZ in PBS into both the ipsilateral and contralateral thigh muscles. This resulted in robust light emission from the ipsilateral leg (peak radiance of ∼150,000 p/s/cm^2^/sr), whereas DTZ injection into the contralateral thigh muscle failed to induce any light emission (Fig. 6*B*).

While BLI is a powerful tool, we have no data on whether injected DTZ is capable of reaching tissues beyond the site of injection, and thus, the preceding observations do not shed light on whether mRNA expression is greater in the injected leg than in internal organs. To explore this possibility, we sought to use the more traditional technique of tissue excision and immunoblot. However, we first needed to establish the utility and specificity of antibodies for detecting Antares2 protein.

As noted previously, the full-length, ∼70 kDa Antares2 protein has two copies of CyOFP1, a fluorescent protein that was derived from mKate (62, 66) by way of Neptune, mNeptune, and mNeptune2 (https://www.fpbase.org/protein/mkate/). Of these proteins, a commercial antibody was available for the detection of mKate, and we therefore tested its reactivity to proteins present in HEK293 cells and in HEK293 cells transfected with plasmids designed to express Antares2-2a or Antares2-myc. These proteins carry carboxy-terminal extensions of 34 or 14 amino acids in the cases of Antares2-2a or Anatres2-myc, respectively. Two days later, the cells were lysed, the resulting protein extracts were separated by SDS-PAGE, transferred to PVDF membranes, and processed for immunoblot using antibodies specific for mKate and HSP90 (Fig. 6*C*). These experiments revealed that the anti-mKate antibodies were specific for Antares2, and yet recognized multiple Antares2 protein products, several of which were larger than the 70 kDa Antares2 predicted molecular mass, and several of which were smaller. Thus, this mKate antibody is specific for Antares2 and moreover, Antares2 migrates as a complex set of immunoreactive species, some of which may be generated by proteolysis while others are likely to represent SDS-resistant oligomers of Antares2 and Antares2 proteolytic fragments.

This complexity of immunoreactive Antares2 bands was also observed in tissue extracts of injected mice. Specifically, mice were injected i.m. with Antares2 mRNA-loaded exosomes, followed 2 days later by excision of tissues and processing of tissue protein lysates by immunoblot (Fig. 6*D*). Encouragingly, the anti-mKate antibody detected three proteins in the injected muscle tissue, one of the expected sizes of Antares 2 (∼70 kDa) and two of higher molecular mass (∼90 kDa and ∼120 kDa). None of these proteins were in either the skeletal muscle of the control mouse or in other tissues of either experimental or control animals, even though all tissues contained mouse protein, as shown by the anti-HSP90 immunoblot.

### Sustained expression upon repeat dosing with mRNA-loaded exosomes

**Figure 7:**
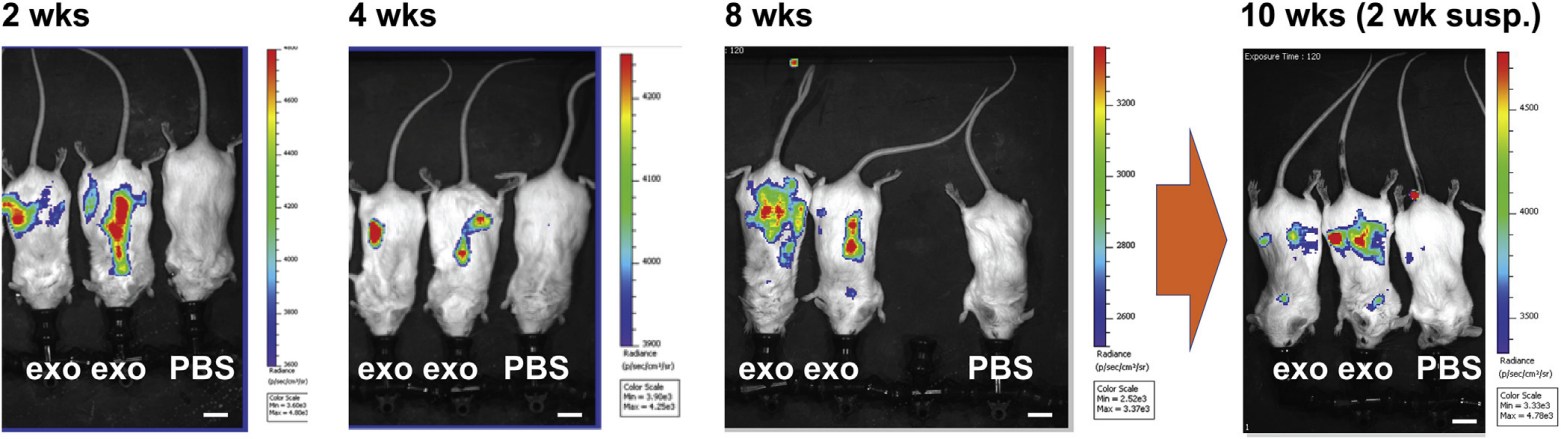
**Repeat dosing of mRNA-loaded exosomes leads to sustained mRNA expression.** Combined light and bioluminescence images of mice that had been injected with 0.05 ml of mRNA loaded exosomes: (2 weeks) on days 0, 7, and 14, and imaged 1 day after the day 14 injection; (4 weeks) on days 0, 7, 14, 21, and 28, and imaged 1 day after the day 28 injection; (8 weeks) on days 0, 7, 14, 21, 28, 42, and 56 and imaged 2 days after the day 56 injection; and (10 weeks) on days 0, 7, 14, 21, 28, 42, and 56 and imaged 16 days after the day 56 injection. Bar, 1 cm. Light emission measured in radiance (photons/s/area (cm^2^)/steradian) is reflected in the color, with *red* denoting the areas of highest light emission and *blue* denoting the areas of lowest light emission. Five treated animals in total were used in these experiments.

Previous studies have established that repeat dosing of nucleic acid delivery systems can lead to attenuation of gene expression and a fall-off in functional gene delivery (67, 68). To explore this issue in the context of exosome-mediated mRNA delivery, we measured Antares2 expression over the course of repeated weekly and biweekly administrations of mRNA-loaded exosomes. Specifically, mice were injected i.m. with 0.05 ml of mRNA-loaded exosomes at weeks 0, 1, 2, 3, 4, 6, and 8. Furthermore, these injections were in all cases followed 1–3 days later by i.p. injection of DTZ solution, with Antares2-mediated light emission detected by BLI. In these experiments, DTZ-induced, Antares2-dependent light emission was detected only in the torso, indicating that DTZ is most accessible to Antares2 where it is administered and is not a faithful reporter of the biodistribution of Antares2 mRNA expression (Fig. 7). Furthermore, the level of light emission in these experiments was ∼30-fold lower than what we had observed in the eye and muscle experiments described previously in Figure 6. Nevertheless, this admittedly imperfect experimental design still allowed us to ask whether repeated administration of mRNA-loaded exosomes might lead to an attenuation in mRNA expression. It did not, and the constancy of light emission is shown here for animals imaged at 2, 4, and 8 weeks after 3, 6, and 7 injections of Antares2 mRNA-loaded exosomes, respectively (Fig. 7). Furthermore, when animals that had been injected six times over the course of 8 weeks were imaged 14 days later (2 weeks after the final exosome injection at week 8), Antares2 expression was still detectable (Fig. 7), demonstrating that sustained, repeated injection of mRNA-loaded exosomes can lead to sustained expression of the encoded protein, even weeks after the final administration.

### Design and validation of S^W1^ and LSNME mRNAs

We next tested whether exosomes could be used to deliver functional mRNAs of clinical importance. We therefore synthesized two mRNAs, one designed to encode S^W1^, the Spike encoded by the wild-type strain of SARS-CoV-2 (23), and another designed to encode LSNME, a fusion protein in which SARS-CoV-2 Nucleocapsid protein and fragments of the Spike, Membrane, and Envelope proteins are expressed in fusion to the human Lamp1 protein. The rationale for including a Spike-encoding mRNA is based on the extensive evidence that this is critical to vaccine efficacy, while the rationale for including Nucleocapsid (and other proteins) is based on the extensive evidence that Nucleocapsid-based vaccines can also protect against various aspects of SARS-CoV-2 infection (44, 51, 52, 69, 70). As for why we chose to express Nucleocapsid and other viral antigens as a Lamp1 fusion protein, previous studies have provided ample evidence that antigen expression as Lamp1 fusions can lead to stronger cellular immune responses than other approaches to antigen expression (51, 71).

**Figure 8:**
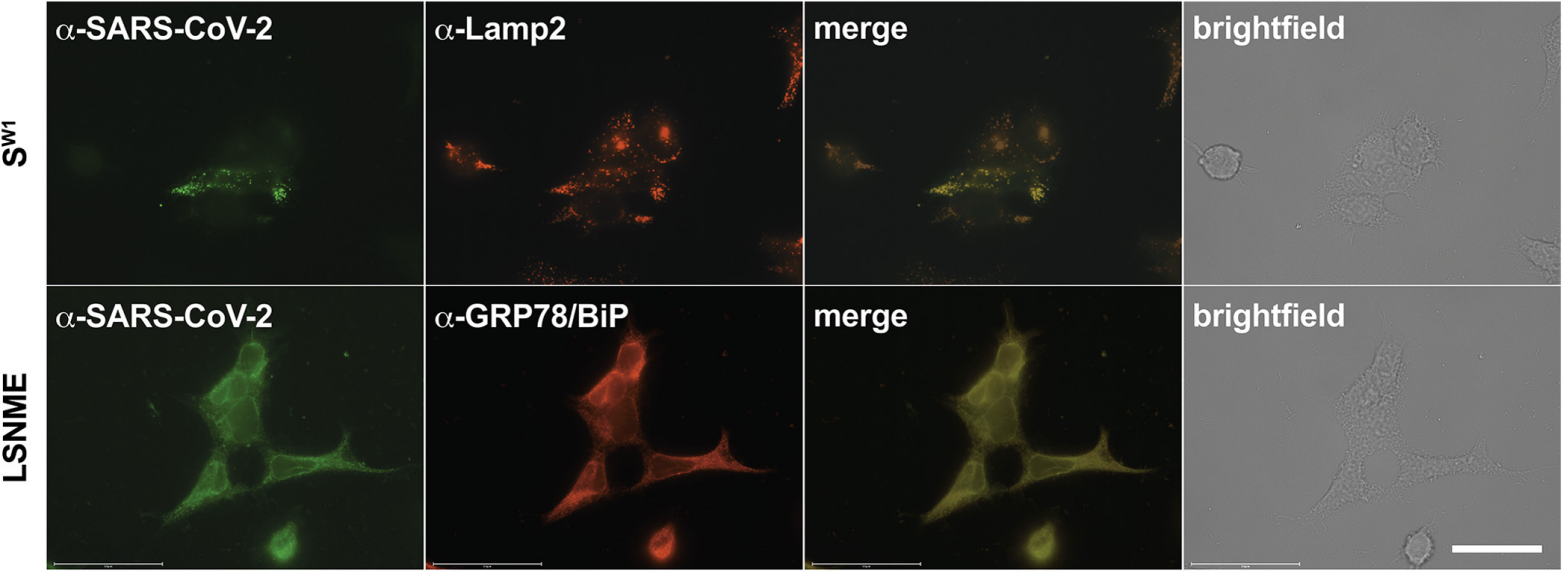
**Subcellular distribution of S**^**W1**^**and LSNME proteins.***Upper panels*, fluorescence and brightfield micrographs of S^W1^ mRNA-expressing HEK293 cells at 2 days after transfection, stained with (*green*) COVID-19 patient plasma and (*red*) an antibody specific for the lysosomal protein Lamp2, with (merge) colocalization evident in *yellow*, and the brightfield image showing all cells in the field. DAPI and a plasma from a COVID-19 patient. *Lower panels*, fluorescence and brightfield micrographs of LSNME mRNA-expressing HEK293 cells at 2 days after transfection, stained with (*green*) COVID-19 patient plasma and (*red*) an antibody specific for the ER protein GRP78/BiP, with (merge) colocalization evident in *yellow*, and the brightfield image showing all cells in the field. Bar, 50 μm.

To ensure that these mRNAs encode proteins that can be expressed in human cells, HEK293 cells expressing these proteins were interrogated by immunofluorescence microscopy (Fig. 8). Using a plasma from COVID-19 patients that is known to contain anti-Spike and anti-Nucleocapsid antibodies (72), we detected Spike in Lamp2-containing lysosomes (Fig. 8*A*), which we previously established to be the primary site of Spike protein trafficking in human cells (72). As for LSNME, it was detected in the ER, where it colocalized with ER protein chaperone GRP78/BiP (Fig. 8*B*).

### The LSNME/S^W1^ vaccine induced anti-N and anti-S antibody responses

**Figure 9:**
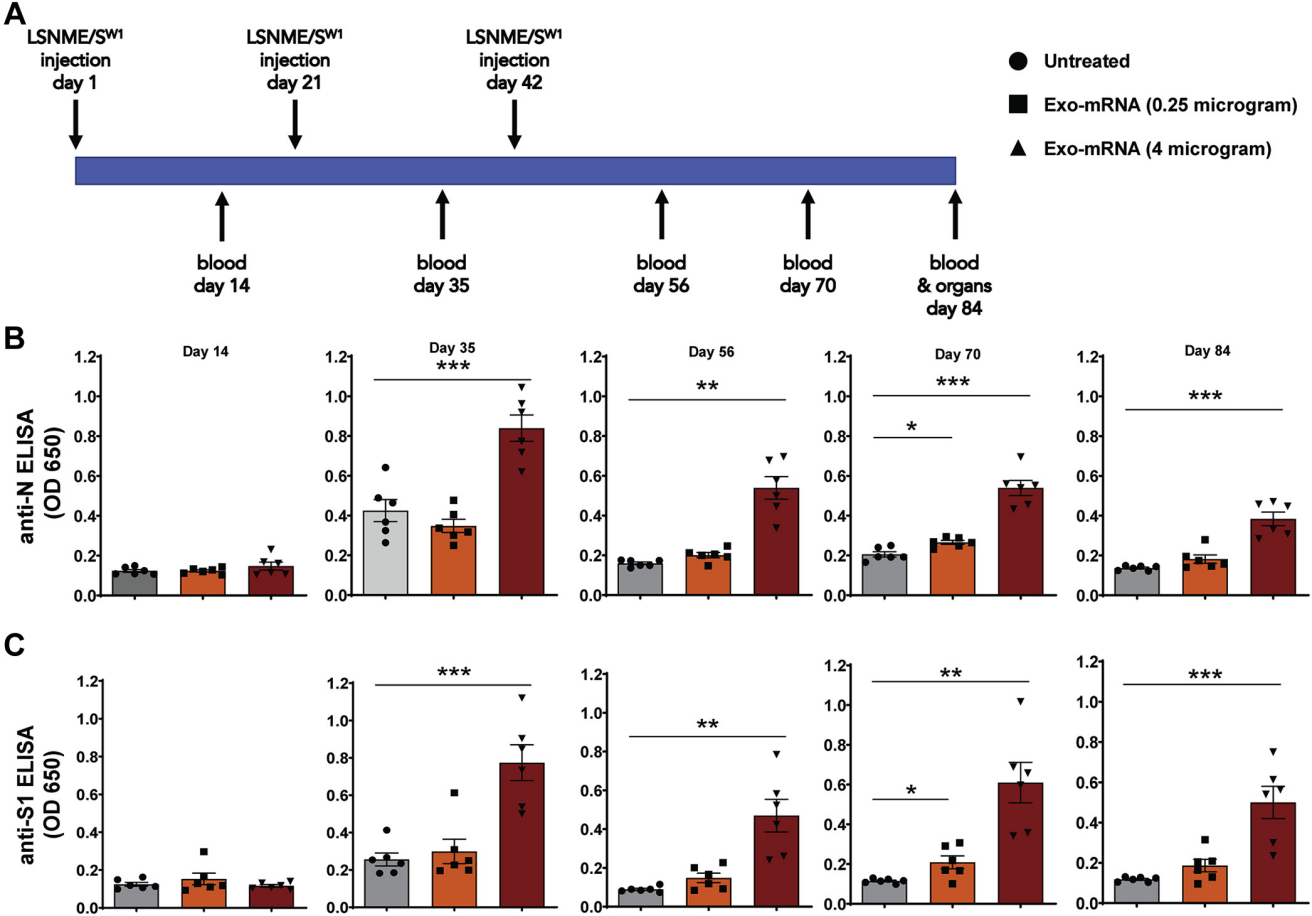
**LSNME/S**^**W1**^**vaccination induces antibody responses to SARS-CoV-2 N and S protein.***A*, schematic of immunization and blood/tissue collection timeline. *B*, anti-N ELISA results of diluted plasma from (*gray bars* and *black circles*) individual six control mice, (*orange bars* and *black squares*) six mice immunized with 0.25 μg equivalents of each mRNA, and (*rust bars* and *black triangles*) six mice immunized with 4 μg equivalents of each mRNA. *C*, anti-S1 ELISA results of diluted plasma from (*gray bars* and *black circles*) individual six control mice, (*orange bars* and *black squares*) six mice immunized with 0.25 μg equivalents of each mRNA, and (*rust bars* and *black triangles*) six mice immunized with 4 μg equivalents of each mRNA. Height of bars represents the mean, error bars represent ± one standard error of the mean, and the statistical significance of differences between different groups is reflected in one-way ANOVA values denoted by ∗ for <0.05, ∗∗ for <0.01, and ∗∗∗ for <0.001.

With their expression confirmed, mRNAs encoding S^W1^ and LSNME were loaded into 293F-derived exosomes and injected (i.m.) into the leg muscle of 13 weeks-old male C57BL/6J mice (Fig. 9*A*). Blood (0.1 ml) was collected on days 14, 35, 56, 70, and 84. On day 84, the animals were sacrificed to obtain tissue samples for histological analysis and splenocytes for blood cell studies. Using ELISA kits adapted for the detection of mouse antibodies, we observed that vaccinated animals displayed a dose-dependent antibody response to both the SARS-CoV-2 Nucleocapsid and Spike proteins (Fig. 9*B*). These antibody reactivities were relatively constant and long-lasting, persisting to the end of the 3-month-long study. It should be noted that modest antibody production was expected in the case of the N protein, as the LSNME mRNA is designed primarily to stimulate cellular immune responses rather than the production of anti-Nucleocapsid antibodies.

### LSNME/S^W1^ vaccination-induced T cell responses to N and S

**Figure 10:**
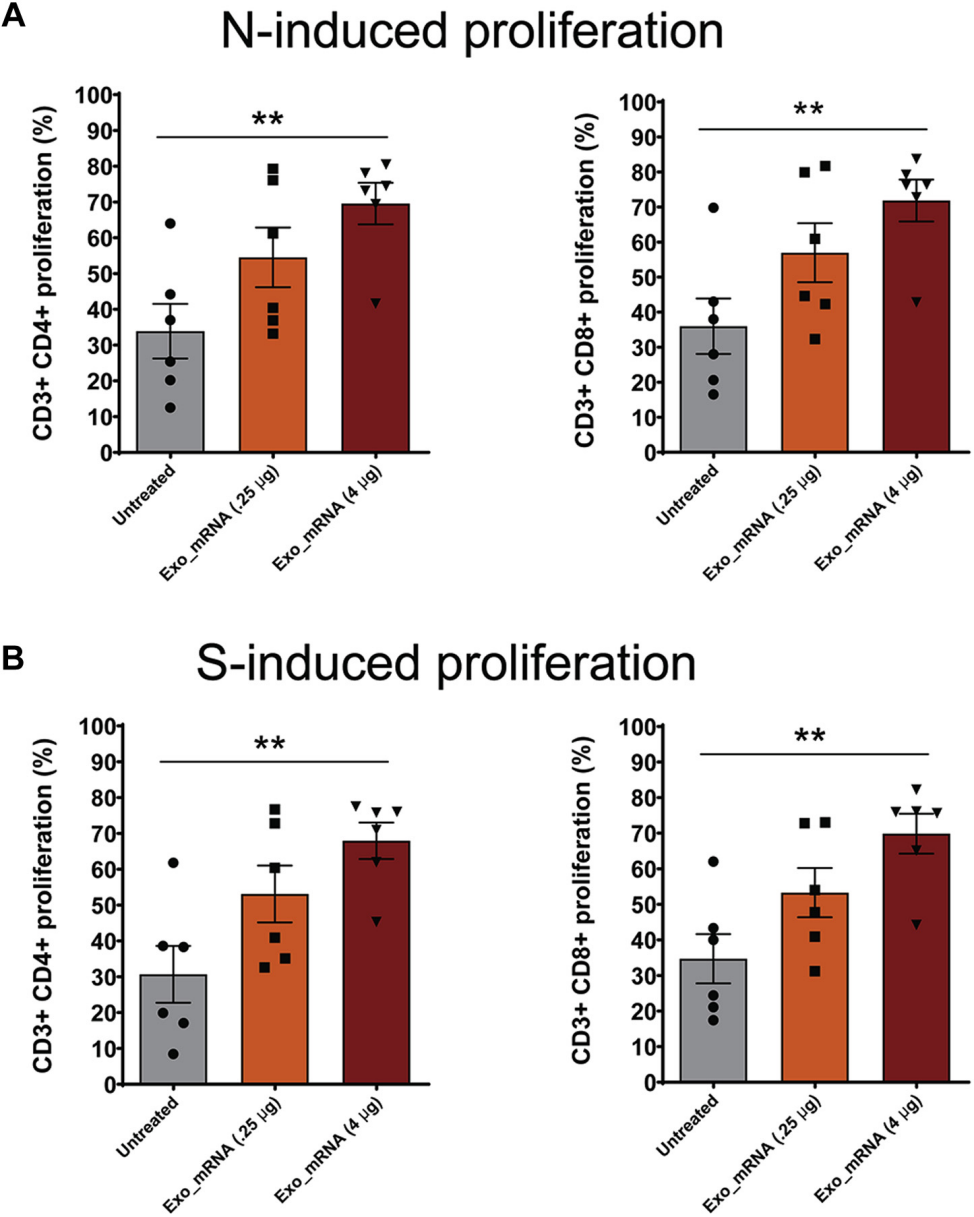
**LSNME/S**^**W1**^**vaccination induces CD4**^**+**^**and CD8**^**+**^**T-cell responses.** CFSE-labeled splenocytes were interrogated by flow cytometry following incubation in the absence or presence of (*A* and *B*) purified, recombinant N protein or (*C* and *D*) purified, recombinant S protein, and for antibodies specific for CD4 and CD8. Differences in proliferation of CD4^+^ cells and CD8^+^ cells were plotted for (*gray bars* and *black circles*) individual six control mice, (*orange bars* and *black squares*) six mice immunized with 0.25 μg equivalents of each mRNA, and (*rust bars* and *black triangles*) six mice immunized with 4 μg equivalents of each mRNA. Height of bars represents the mean, error bars represent ± one standard error of the mean, and the statistical significance of differences between different groups is reflected in one-way ANOVA values denoted by ∗ for <0.05 and ∗∗ for <0.01.

**Figure 11:**
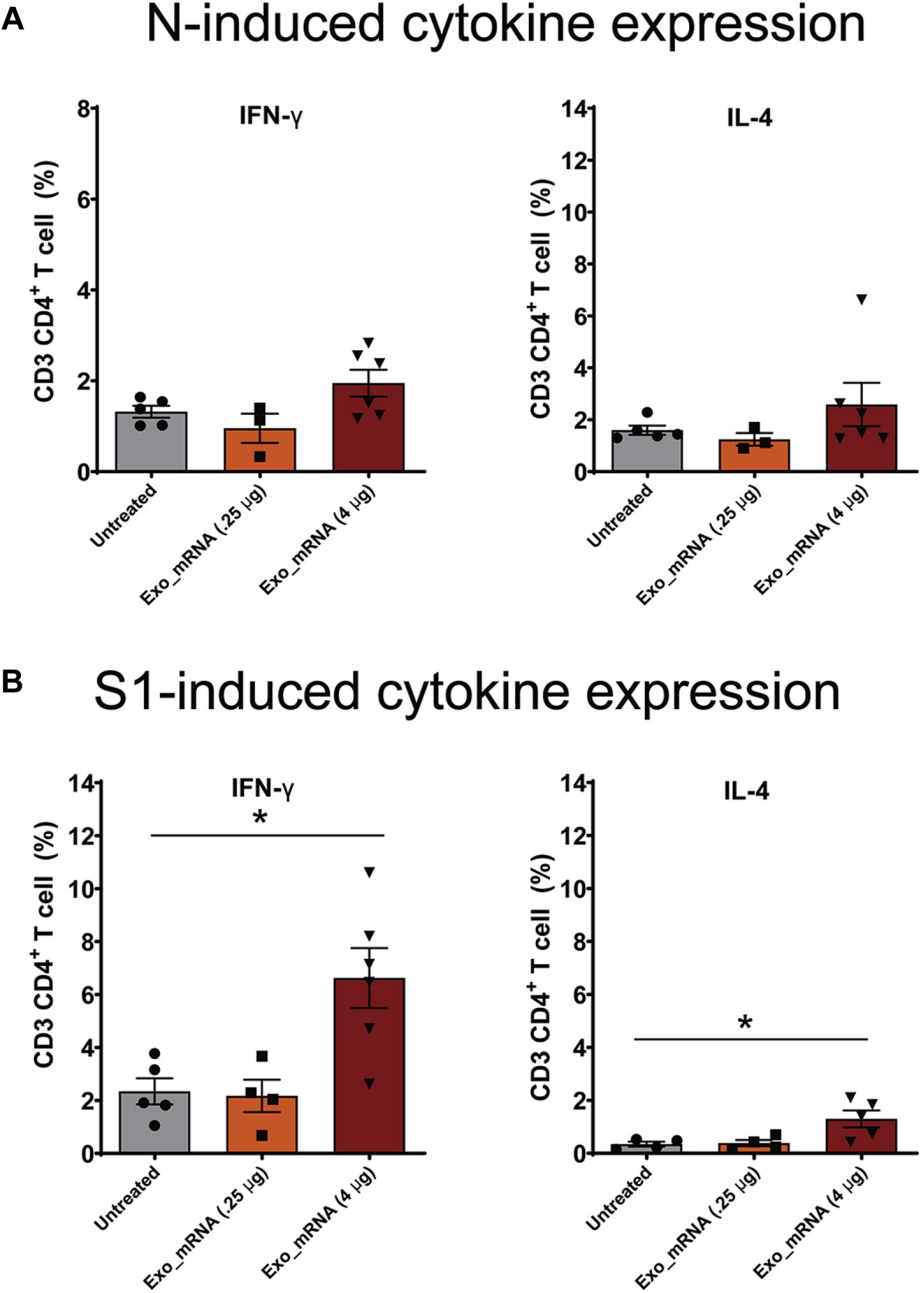
**LSNME/S**^**W1**^**vaccination leads to S-induced expression of IFNγ and IL4 by CD4**^**+**^**T-cells.** Splenocytes were interrogated by flow cytometry following incubation in the absence or presence of (*A* and *B*) purified, recombinant N protein or (*C* and *D*) purified, recombinant S protein, and labeling with antibodies specific for CD4 or CD8, and for IFNγ or IL4. Differences in labeling for IFNγ or IL4 in CD4^+^ CD8^+^ cell populations were plotted for (*gray bars* and *black circles*) individual six control mice, (*orange bars* and *black squares*) six mice immunized with 0.25 μg equivalents of each mRNA, and (*rust bars* and *black triangles*) six mice immunized with 4 μg equivalents of each mRNA. Height of bars represents the mean, error bars represent ± one standard error of the mean, and the statistical significance of differences between different groups is reflected in one-way ANOVA values, with ∗ denoting <0.05.

Vaccinated and control animals were also interrogated for the presence of antigen-reactive CD4+ and CD8+ T-cells. This was carried out by collecting splenocytes at the completion of the trial (day 84) and performing a CFSE proliferation assay in the presence or absence of recombinant Nucleocapsid and Spike proteins. These experiments revealed that vaccination induced a significant increase in the percentage of CD4^+^ T-cells and CD8^+^ T-cells that grew in response to adding recombinant N and S proteins to the culture media (Fig. 10, *A–D*). These vaccine-specific, antigen-induced proliferative responses demonstrate that the LSNME/S^W1^ vaccine achieved its primary goal, which was to prime the cellular arm of the immune system against the Spike and Nucleocapsid proteins. Antigen-induced T-cells cells were also tested for the expression of interferon gamma (IFNγ) and interleukin 4 (IL4), revealing that the Spike-reactive CD4^+^ T-cell population displayed elevated expression of the Th1-associated cytokine IFNγ, and to a lesser extent, the Th2-associated cytokine IL4 (Fig. 11). In contrast, N-reactive T-cells failed to display an N-induced expression of either IFNγ or IL4.

### Exosome-based LSNME/S^W1^ vaccination caused no adverse reactions in immunized animal models

**Figure 12:**
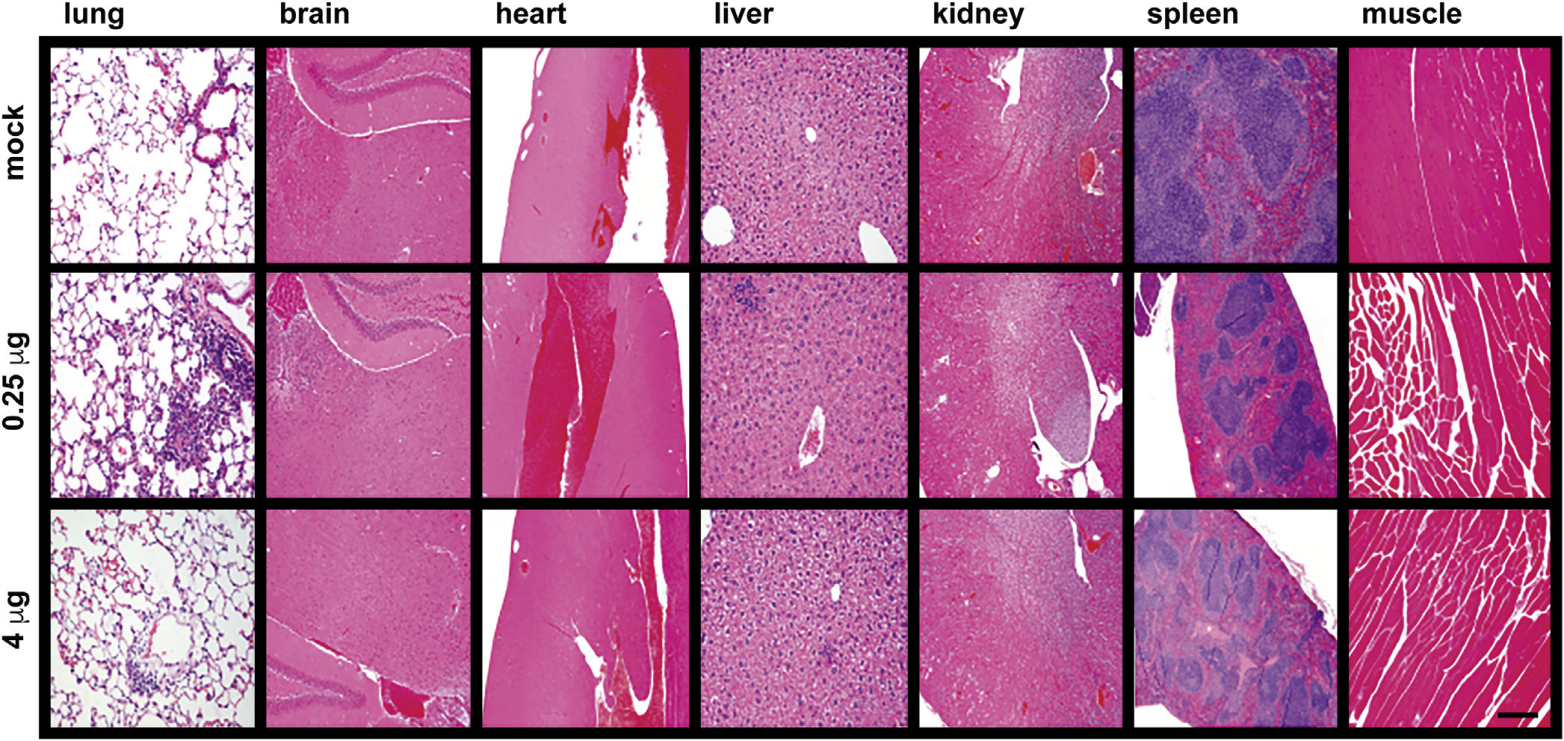
**Absence of tissue pathology upon LSNME/S**^**W1**^**vaccination.** Representative micrographs from histological analysis (hematoxylin and eosin stain) of the lung, brain, heart, liver, kidney, spleen, and muscle (side of injection) of animals from (*upper row*) control mice, (*middle row*) mice immunized with the lower dose of the LSNME/S^W1^ vaccine, and (*lower row*) mice immunized with the higher dose of the LSNME/S^W1^ vaccine. Bar, 0.2 mm.

Control and vaccinated animals were examined regularly for overall appearance, general behavior, and injection site inflammation (redness, swelling). No vaccine-related differences were observed in any of these variables, and animals from all groups displayed similar age-related increases in body mass (Fig. S3). Vaccination also had no discernable effect on blood cell counts (Fig. S4). Histological analyses were performed by an independent histology service (University of Washington) on all animals at the completion of the study, which concluded that the vaccinated animals showed no test-article-related differences in any of the tissues in any of the animals. Representative images are presented here for the brain, lung, heart, liver, spleen, kidney, and skeletal muscle in an animal from each of the trial groups (Fig. 12).

## Discussion

### 293F-derived exosomes are safe and nontoxic

Exosome exchange occurs during normal human interactions such as breastfeeding and sex, is intrinsic to all tissue transplantations, blood transfusions, and plasma injections, and is the objective of clinic-based therapies involving exosomes derived from amniotic fluid, cord blood, and other natural human biofluids (73, 74). In each of these settings, exosome exchange appears to be safe and well tolerated. Our study adds to this story by showing that exosomes had no adverse effects on cells *in vitro* or *in vivo*. This cannot be said of LNPs, which were already known to have adverse effects (10, 53, 54) and were found in this study to cause pronounced cellular toxicity.

These results warrant a brief discussion of why exosomes are so benign. One likely reason is that exosomes have been continuously present in all extracellular fluids/biofluids of all animals, throughout the evolution of multicellular life and throughout the lifetime of all individuals (12). In other words, they are the only bionormal nanovesicle, more self than non-self, and therefore well recognized as a normal constituent of the mammalian body. A second reason they are so well tolerated is likely related to the fact that they are extraordinarily small, with a diameter ∼1/10th the wavelength of light and a volume ~1/10,000,000th of a human cell. As a result, exosome injections simply do not deliver large quantities of proteins or lipids that might otherwise stimulate strong inflammatory or immune reactions.

### Creation and characterization of mRNA-loaded exosomes

The bionormal character of exosomes indicates that they can be used as safe and nontoxic nanoscale delivery vehicles for mRNAs and other drugs. This hypothesis is supported by our observation that exosomes can be loaded with RNA at encapsulation efficiencies that exceed 90%, without noticeable degradation of input mRNA or gross alteration of exosome size and concentration. This process results in the creation of exosomes that contain high levels of the desired mRNA while preserving their bionormal characteristics. Not surprisingly, these mRNA-loaded exosomes were as or more effective at delivering functional mRNA into cells as mRNA-loaded LNPs and were far more effective than mRNA-loaded LNPs at high particle concentrations.

### Exosomes can mediate localized mRNA delivery

Our results also demonstrated that mRNA-loaded exosomes can generate localized mRNA expression following injection into either the eye or skeletal muscle. These results raise the possibility that localized injection of mRNA-loaded exosomes can be used to deliver therapeutic mRNAs to where they are needed. In the eye, intravitreal injection has the potential to reach many different cell types and perhaps even the retinal pigment epithelium (RPE) (75) that is impaired in retinitis pigmentosa and several other genetic disorders of the visual system. It will be exciting to see if similar results can be obtained for delivery to the brain (*via* intrathecal administration), the respiratory tract (*via* nasal/bronchial administration), joints (*via* intrasynovial administration), or the placenta/amnion (*via* intraamniotic administration).

Although i.m. injection of Antares2 mRNA-loaded exosomes resulted in pronounced expression in injected muscle, it also resulted in a low level of systemic Antares2 expression elsewhere in the body. This extramuscular expression of Antares2 was undetectable by immunoblot but could be observed by BLI following i.p. injection of DTZ. This property not only allowed us to explore the repeatability of mRNA/exosome injections, but establishes that exosomes can also be delivered systemically following a localized injection, a result that bodes well for use of mRNA/exosome formulations in therapies that require repeated mRNA injections over extended periods of time. This result anticipates a wide variety of possible studies on systemic injection of mRNA-loaded exosomes, not only to deliver mRNAs throughout the body, but also to deliver mRNAs to areas of chronic inflammation and cancer that appear to concentrate systemically injected exosomes (20, 21).

### Exosome-based SARS-CoV-2 mRNA delivery and immunogenicity

We observed that i.m. injection of mRNA-loaded exosomes leads to far higher mRNA expression at or near the site of injection than in internal organs. This finding bodes well for the development of exosome-delivered mRNA vaccines, as it suggests that the cytotoxic effects arising from boost injections will be localized primarily to the skeletal muscle, which has a high functional tolerance to damage and a strong regenerative capacity. Consistent with this hypothesis, immunization of mice with mRNA-loaded exosomes had no adverse effects on experimental animals. Immunization of mice with mRNA-loaded exosomes did, however, induce dose-dependent development of anti-Spike and anti-Nucleocapsid antibody responses as well as antigen-specific CD4^+^ and CD8^+^ T-cell responses. These immune responses persisted for months. Moreover, they were associated with a Th1 cytokine profile that correlates well with the induction of protective antiviral immunity (large increase in IFNγ, smaller increase in IL-4).

These results represent the first preclinical test of mRNA-loaded exosomes, demonstrate that this platform can successfully deliver multiple functional mRNAs and support the further development of mRNA-loaded exosomes for use as vaccines and therapeutics.

## Experimental procedures

### Cell lines and cell culture

HEK293 cells (ATCC CRL-1573) were grown in DMEM supplemented with 10% fetal bovine serum (FBS) and 1% pen/strep solution. 293F cells (Thermo Fisher A14528) were tested for pathogens and found to be free of viral (cytomegalovirus, human immunodeficiency virus I and II, Epstein Barr virus, hepatitis B virus, and parvovirus B19) and bacterial (*Mycoplasma*) contaminants. Cells were maintained in FreeStyle 293 Expression Medium (Gibco, 12338-018) and incubated at 37 °C in 8% CO_2_. For exosome production, 293F cells were seeded at a density of 1.5 × 10^6^ cells/ml in shaker flasks in a volume of ∼1/4 the flask volume and grown at a shaking speed of 110 rpm. For cultures used in luciferase assays and MTT assays, 293F cells were grown in DMEM supplemented with 10% fetal calf serum and 1% penicillin and streptomycin solution. Human Umbilical Vein Endothelial cells (HUVEC; Thermo Fisher C0035C) and Human Dermal Fibroblast cells (HDF; GIBCO C-013-5C) were grown in Medium 200 (Thermo Fisher M200-500) or Medium 106 (Thermo Fisher M106-500) supplemented with low serum growth supplement (LSGS; Thermo Fisher S-003-10) at 37 °C and 5%CO_2_. Cells were transfected with plasmids using Lipofectamine 3000, or mRNA using Lipofectamine Messenger MAX, as suggested by the manufacturer (Thermo Fisher). Antares2 expression vectors were pcDNA3-Antares2myc (Addgene 100027) or pJM1333 (Antares2-2a), which carried the Antares2 ORF immediately upstream of the viral 2a peptide and PuroR gene in plasmid pJM1242 (76).

### Exosome purification

293F cells were grown in Freestyle media as described above for a period of 3 days. Cells were removed by centrifugation at 300*g* for 5 min, and large cell debris was removed by centrifugation at 3000*g* for 15 min. This generated the conditioned media that was passed through an ∼200 nm pore size diameter sterile filtration unit to generate a CTCS. The CTCS was concentrated by centrifugal filtration (Centricon Plus-70, MilliporeSigma), with ∼120 ml CTCS yielding a concentrated vesicle supernatant (CVS) of ∼0.5 ml. Vesicles in the CVS were then separated from free protein by SEC, using PBS as column buffer (qEV column, Izon). Exosomes eluting in the postvoid fractions and the three peak exosome fractions were pooled. This pool of peak exosome-containing fractions was purified further by another round of centrifugal filtration (Ultra-4, 100 kDa cutoff, Amicon), generating the purified exosome preparation.

### Molecular measurements and enzyme assays

Total protein was determined by bicinchoninic acid (BCA) assay of detergent-solubilized samples (ThermoFisher 23225). Lipid were extracted from cell and exosome samples using the Folch Method and quantified using a commercial assay kit (Abcam ab242305). DNA was extracted using a DNeasy Blood & Tissue kit (69506, Qiagen), RNA was extracted using a RNEasy Mini Plus kit (Qiagen 74136), and resulting DNA and RNA samples were interrogated using a bioanalyzer 2100 (Agilent). The MTT assay was performed by incubating cells with 0.2 ml of 0.5 mg/ml 3-(4,5-dimethylthiazol-2-yl)-2,5-diphenyltetrazolium bromide (MTT) in PBS for 4 h at 37 °C in the dark. The MTT solution was removed, 0.2 ml of acidic isopropanol (0.1 N HCL/100% isopropanol) was added, the plates were incubated for another hour at 37 °C in the dark and read for absorbance at 570 nm in a plate reader

### Nanoparticle tracking analysis (NTA)

Vesicle concentrations and sizes were measured by NTA using a PMX-220 ZetaView camera (Particle Metrix) with samples diluted in particle-free PBS (pfPBS), which PBS clarified immediately prior to use by 100 nm pore size filtration (to minimize the presence of nanometer scale phosphate crystals). NTA measurements were carried out in triplicate at ambient temperature with fixed camera settings. Fluorescent nanoparticle tracking analysis (flNTA) was performed by incubating Alexa Fluor 488-conjugates of monoclonal anti-CD63 antibodies with exosome suspensions (2 h, RT, in absence of light), diluting them in pfPBS, and then interrogating them using a PMX-220 ZetaView camera (Particle Metrix). Samples were visualized in scatter mode using the 488 nm laser and standard instrument settings in fluorescence mode with standard fluorescence settings. Imaging data was analyzed with the ZetaView software 8.05.10 (Particle Metrix).

### Immunoblots

Exosome and cell lysates were separated by SDS-PAGE using precast, 4–15% gradient gels and transferred to PVDF membranes. Membranes were blocked for 2 h using 5% nonfat dry milk in Tris-buffered saline (TBS) with Tween-20 (TBST; 138 mM NaCl, 2.7 mM KCl, 50 mM Tris, pH 8.0, 0.05% Tween-20). Membranes were then incubated overnight in blocking solution containing antibodies obtained from commercial sources (CD9 [clone HI9a, BioLegend]; CD63 [clone MX-49.129.5, Santa Cruz Biotechnology], CD81 [555675, BD Pharmingen], E-cadherin [20874-1-AP, Thermo Fisher]; N-cadherin [MA1-2002, Thermo Fisher]; GRP78/BiP [PA1-014A, Thermo Fisher]; calreticulin [MA5-32131, Thermo Fisher]; ERGIC-3 [clone B5, sc-398778, Santa Cruz Biotechnology]; GM130 [PA1-077, Thermo Fisher]; HSP60 [ab190828, Abcam]; HSP90 [sc-13119; Santa Cruz Biotechnology]; Lamp2 [MA1-205, Thermo Fisher]). The next day, the membranes were washed, exposed to HRP-conjugates of goat secondary antibodies (Jackson Immunoresearch), washed again, incubated with an HRP-activated chemiluminescence detection solution (Amersham), and imaged using a GE Amersham Imager 600. Images were exported as JPEG files and assembled into figures using Adobe Illustrator and Adobe Photoshop.

### Electron microscopy and light microscopy

Exosomes were fixed by addition of formaldehyde to a final concentration of 4%. Carbon-coated grids were placed on top of a drop of the exosome suspension. Next, grids were placed directly on top of a drop of 2% uranyl acetate. The resulting samples were examined with a Tecnai-12 G2 Spirit Biotwin transmission electron microscope (John Hopkins University). For immunofluorescence microscopy, HEK293 cells were transfected with LSNME or Spike expressing plasmids, then grown on poly-L-lysine-coated sterile coverglasses for 24 h. Cells were then fixed using 3.7% formaldehyde in PBS, permeabilized in 1% Triton X-100 in PBS, colabeled with COVID-19 patient plasma (1:1000 dilution) and antibodies specific for either Lamp2 or GRP78/BiP, washed, incubated with Alexa Fluor 488-labeled donkey anti-human IgG and Alexa Fluor 647-labeled donkey anti-mouse or anti-rabbit IgG (Jackson ImmunoResearch; 1:1000 dilution), washed, followed by mounting of coverglasses on glass slides. Cells were visualized using an EVOS M7000 microscope. Digital images were acquired as PNG or TIF files, modified in Photoshop (Adobe), and figures were assembled using Illustrator (Adobe). Patient plasmas were collected using standard procedures for blood draw and plasma collection. After Johns Hopkins Medicine Institutional Review Board (IRB) approval, plasma samples were obtained under informed consent from healthy donors before the COVID-19 pandemic (JHM IRB NA_0004638) and from patients with COVID-19, with specimens utilized for this publication obtained from the Johns Hopkins Biospecimen Repository, which is based on the contribution of many patients, research teams, and clinicians, which were collected after IRB approval (Johns Hopkins COVID-19 Clinical Characterization Protocol for Severe Infectious Diseases [IRB00245545] and Johns Hopkins COVID-19 Remnant Specimen Repository [IRB00248332]).

### Production of mRNA-loaded exosomes and LNPs

mRNAs designed to express the Antares2, S^W1^ and LNSME proteins were obtained from a commercial provider (Trilink). mRNAs were purified using RNeasy columns (Qiagen) and resuspended in DNase-free, RNase-free water using nuclease-free tips and tubes. Purified mRNAs were prepared for loading into exosomes or LNPs by preincubating them with a solution of cationic lipids, generating lipid-coated mRNAs. Lipid-coated mRNAs were subsequently loaded into purified exosomes or into LNPs (DOTAP/DOPE, #F50102, FormuMAx Scientific Inc) by mixing and incubation.

### Measurement of mRNA encapsulation

The efficiency of mRNA loading was determined by separating free Antares2 mRNA, mRNA-loaded exosomes, and lysates of mRNA-loaded exosomes using the RNA chip on an Agilent bioanalyzer, with the amount of RNA detected reflecting the input and nonencapsulated RNA amounts, which averaged >90%. The same technique was used to interrogate the extractable mRNA from mRNA-loaded exosomes, which demonstrated that it had not been degraded by the loading process. The incorporation of RNA into exosomes by fluorescence was carried out using the Quant-iT RiboGreen RNA Assay Kit (ThermoFisher). In brief, RNA standards and experimental samples were mixed with the RiboGreen dye, and sample fluorescence was measured (excitation at 485 nm, emission at 535 nm). Calibration standards of known quantities of bacterial 16S and 23S rRNAs were used to generate a standard curve from which sample RNA concentrations were deduced. Experimental samples were equal amounts, by input mRNA, of (a) pure mRNA, (b) RNA following incubation with, and coating by, cationic lipid, and (c) the final mRNA-loaded exosomes. To determine whether the mRNA that had been encapsulated into exosomes was intact, mRNA-loaded exosomes were subjected to RNA extraction followed by interrogation using a bioanalyzer and RNA analysis chip, as recommended by the manufacturer (Agilent).

### Animal experimentation

All animal experimentation was performed following institutional guidelines for animal care, under the oversight of, and approval by, the Cedars-Sinai Medical Center (IACUC #8602). Experiments involved injection of exosomes, LNPs, mRNA-loaded exosomes or mRNA-loaded LNPs into either BALB/c or C57BL/6J mice (Jackson Laboratory). For studies designed to assess the physiological consequences of exosome injections, age-matched BALB/c mice (5 each group and timepoint) were anesthetized using isoflurane and injected (i.m.) with 0.050 ml of PBS, exosomes, LNPs, mRNA-loaded exosomes or mRNA-loaded LNPs on day 0, or on day 0 and again on day 14. Animal masses were determined periodically, and blood (∼0.1 ml) was collected periodically from the orbital vein. At dates specified by our research protocol (day 3 and day 28), animals were deeply anesthetized using isoflurane, euthanized by cervical dislocation, and processed using standard surgical procedures to obtain the intestine, salivary glands, spleen, kidney, liver heart, diaphragm, lung, and brain, which were processed for histological analysis by fixation in 10% neutral buffered formalin. Histological analysis was performed by the service arm of the HIC/Comparative Pathology Program of the University of Washington, with results documented by image capture and summarized in the pathologist's report.

For studies designed to interrogate functional mRNA expression in the eye, BALB/c mice were anesthetized using isoflurane and injected intravitreally with 0.0020 ml of either PBS or Antares2 mRNA-loaded exosomes. Thirty-six hours later, the animals were again anesthetized using isoflurane and the injected eye was exposed to a drop of 0.010 mM DTZ in PBS, and the mice were examined by BLI. For studies designed to interrogate functional mRNA expression in skeletal muscle, a single BALB/c mouse was anesthetized using isoflurane and injected i.m in the left thigh with 0.0050 ml of either PBS or Antares2 mRNA-loaded exosomes. Forty-eight hours later, the animal was again anesthetized using isoflurane followed by injection (i.m.) of 0.050 ml of 0.010 mM DTZ in PBS in both the ipsilateral and contralateral legs and examination by BLI. For studies designed to interrogate functional mRNA expression in tissues other than the muscle, each BALB/c mouse was anesthetized using isoflurane and injected i.m in the left thigh with 0.0050 ml of either PBS or Antares2 mRNA-loaded exosomes. At 1, 2, 7, or 14 days later, each animal was again anesthetized using isoflurane followed by injection (i.p.) of 0.10 ml of 0.010 mM DTZ in PBS, followed by examination by BLI.

For vaccination studies, age-matched C57BL/6J mice were anesthetized using isoflurane and injected i.m in the left thigh with 0.0050 ml of PBS, a low dose of mRNA-loaded exosomes (0.00025 μg equivalents of each mRNA), or a high dose of mRNA-loaded exosomes (0.004 μg equivalents of each mRNA). These injections were repeated on days 21 and 42. At day 84, animals were deeply anesthetized using isoflurane, euthanized by cervical dislocation, and processed using standard surgical procedures to obtain the intestine, salivary glands, spleen, kidney, liver heart, diaphragm, lung, and brain. Spleens were processed to release splenocytes for subsequent analysis of lymphocyte populations. All tissues were processed for histological analysis by fixation in 10% neutral buffered formalin. Histological analysis was performed by the service arm of the HIC/Comparative Pathology Program of the University of Washington, with results documented by image capture and summarized in the pathologist's report.

### Measurement of Antares2 activity in cell culture and *in vivo*

For *in vitro* assays, 293F cells were incubated with mRNA-loaded exosomes or mRNA-loaded LNPs overnight under standard cell culture conditions. Antares2 luciferase activity was measured by Live cell bioluminescence and was collected after incubating with substrate diphenylterazine (Medchemexpress) at final concentration of 50 μM for 3 min. Readings were collected using SpectraMax i3x (Molecular Devices) or BioTek Cytation 5 microplate readers. For *in vivo* BLI studies we used age-matched, female Balb/c mice (Jackson Laboratory). All animal experimentation was performed following institutional guidelines for animal care and use and was approved by Cedars-Sinai Medical Center (IACUC #8602). For eye studies, animals were anesthetized, followed by injection of 0.002 ml of exosome/mRNA formulation into the vitreous. Thirty-six hours later, each animal was again anesthetized, saline containing DTZ was applied to the eye surface, and the animals were processed for Antares2-mediated bioluminescent imaging (BLI) using an IVIS Spectrum imager (PerkinElmer). For skeletal muscle studies, each animal was anesthetized, followed by intramuscular (i.m.) injection of 0.050 ml of exosome/mRNA formulation into the left thigh. 48 h later, each animal was anesthetized, followed by injection (i.m.) of DTZ solution into both the left leg and the right left leg, and BLI. For distributional BLI, each animal was anesthetized, followed by intramuscular (i.m.) injection of 0.050 ml of exosome/mRNA formulation into either the right or left thigh. Animals were returned to their cages for 1, 2, 7, or 14 days. For BLI, each animal was anesthetized, followed by intraperitoneal (i.p.) injection of DTZ (in PBS) and BLI.

### Spleen lymphocyte isolation and characterization

Spleens from euthanized mice (day 84) were excised, and single cells were obtained by mechanical passage through a 0.040 mm mesh size nylon cell strainer. Erythrocytes were lysed using ammonium chloride potassium (ACK) buffer (Thermo Fisher), splenocytes were collected by centrifugation at 300*g* for 5 min and resuspended in R10 media (RPMI 1640 media (ATCC) supplemented with 10% fetal bovine serum (FBS), 50 μM 2-mercaptoethanol, penicillin/streptomycin (VWR), and 10 mM HEPES). For surface staining, splenocytes (2 × 10^5^ cells) were resuspended in 100 μl of 10% FBS in PBS and incubated for 30 min at 4 °C in the dark with fluorochrome-conjugated antibodies specific for CD3 (Invitrogen, #17-0032-82) CD4 (Biolegend, #100433), CD8 (Biolegend, #100708), B220 (BD, #552771) CD11c (Invitrogen, #17-0114-81), F4/80 (Invitrogen, #MF48004) Ly6G (Invitrogen, #11-9668-80), and Ly6C (BD, #560592). Following incubation, samples were washed twice with 200 μLs 10% FBS in PBS, cells were pelleted by centrifugation at 300*g* for 5 min. Next the cells were fixed with 100 μLs ICS fixation buffer (Thermo Fisher) and interrogated by flow cytometry using a FACS Canto II (BD Biosciences) with cell counts of 2000–10,000 recorded lymphocytes per sample. Data was analyzed using FlowJo 10 software (FlowJo, LLC).

### Antigen-specific T cell proliferation measurements

Splenocytes were resuspended at 10^6^ cells/ml in 10% FBS in PBS and stained with carboxyfluorescein succinimidyl ester (CFSE) (Thermo Fisher) by mixing equal volumes of cell suspension with 0.010 mM CFSE in 10% FBS in 1× PBS for 5 min at 37 °C. Cells were then washed three times with R10 complete medium and incubated for 96 h in the presence of medium alone or 0.010 mg/ml SARS-CoV-2 antigens N or S1 (NUN-C5227 and SIN-C52H4, Acro Biosystems). After 96 h, cells were washed with 0.2 ml 10% FBS in PBS and centrifuged at 300*g* for 5 min 2at RT. Cells were then stained with anti-CD3-APC (Thermo Fisher, #17-0032-82), anti-CD4-PerCP-Cy5.5 (Biolegend, #100433), or anti-CD8-PE antibodies (Biolegend, #MCD0801) for 30 min at 4 °C. The stained cells were washed twice with 0.2 ml PBS and analyzed by flow cytometry using a FACS Canto II (BD Biosciences) gated first for CD3+ T-cells and then for CD4+/CD8− or CD8+/CD4− populations. Data was analyzed using FlowJo 10 software (FlowJo LLC).

### Intracellular cytokine staining

2.0 × 10^5^ splenocytes were incubated for 72 h in R10 media or R10 media containing of 0.010 mg/ml SARS-CoV2 antigens N or S1 (Acro Biosystems). Cells were washed with fresh R10 medium, incubated with 50 ng/ml phorbol myristate acetate (PMA) (MilliporeSigma, #P1585), 350 ng/ml ionomycin (Thermo Fisher, #124222), and 800 nl/ml GolgiPlug (ThermoFisher, #51-2301KZ) for 4 h to amplify the intracellular accumulation of secretory cytokines. Cells were then washed with 10% FBS in PBS, stained with anti-CD3-APC, anti-CD4-PerCP-Cy5.5, and anti-CD8-PE antibodies (30 min at 4 °C in the dark), washed twice with PBS, fixed, permeabilized, and stained for IFN-γ (eBioscience, #11-7311-82) and IL-4 (Invitrogen, #12-7041-41) overnight at 4 °C in permeabilization buffer. Cells were then interrogated by flow cytometry, with 5000–10,000 recorded cells per sample, and the data was analyzed using FlowJo 10 software.

### ELISA for SARS-CoV-2 antigen-specific antibody responses

Mouse IgG antibody against SARS-CoV-2 antigens was measured by enzyme-linked immunosorbent assays (ELISA) using precoated ELISA plates (IEQ-CoV S RBD-IgG and IEQ-CoVN-IgG, RayBiotech) according to the manufacturer's instructions, at RT. Briefly, mouse plasmas (1:50) were added to preblocked, antigen-coated wells, in duplicate, and incubated at room temperature (RT) for 2 h with gentle rotation (200 rpm). Plates were washed four times with buffer, incubated for 2 h at RT with 1% BSA in PBS, and then with HRP-conjugated goat anti-mouse secondary antibodies (Jackson ImmunoResearch) for 2 h. Wells were washed four times and developed using TMB substrate (RayBiotech). Absorbance at 650 nm was determined for each well using a plate reader (SpectraMaxID3).

### Statistical analysis

Statistical analysis was performed using GraphPad Prism 8 software for Windows/Mac (GraphPad Software) or Excel. Results are reported as mean ± standard deviation or mean ± standard error, and the differences were analyzed using Student's *t* test or one-way analysis of variance.

## Data availability

All data is contained within this paper.

## Supporting information

This article contains supporting information.

## Conflict of interest

S. J. G is a paid consultant for Capricor, holds equity in Capricor, and is coinventor of intellectual property licensed by Capricor. S. J. T. is coinventor of intellectual property licensed by Capricor. C. G. is coinventor of intellectual property licensed by Capricor. N. A. A., J. N., M. C., A. Salehi, A. Sedgwick, S. K., S. S., and C. L. contributed to this work as employees of Capricor.
