# Supplementary material for: Exosome-mediated mRNA delivery in vivo is safe and can be used to induce SARS-CoV-2 immunity
Source: J Biol Chem. 2021 Oct 1;297(5):101266. doi: 10.1016/j.jbc.2021.101266 (PMC8483990; doi:10.1016/j.jbc.2021.101266)

Supporting Information

Figure S1. *Total amount of DNA, RNA, protein, and lipid in cells and exosomes from cultures of 293F cells*. Bar graphs showing the total amount of these four macromolecules in the cells and exosomes of three independent 293F cultures (n = 3). Bar height represents the average and the hatched line shows the standard error of the mean. Cells were seeded at an initial density of 1.5 x 10^6 cells/mL in an average starting volume of 415 mL in 2 L sterile tissue culture flasks, then grown for three days, with shaking. At the conclusion of the culture, cells and exosomes were collected, aliquots of each were interrogated for levels of DNA, RNA, protein, and lipid, and the total amount of each class of molecule in the cell and exosome pellets were calculated.

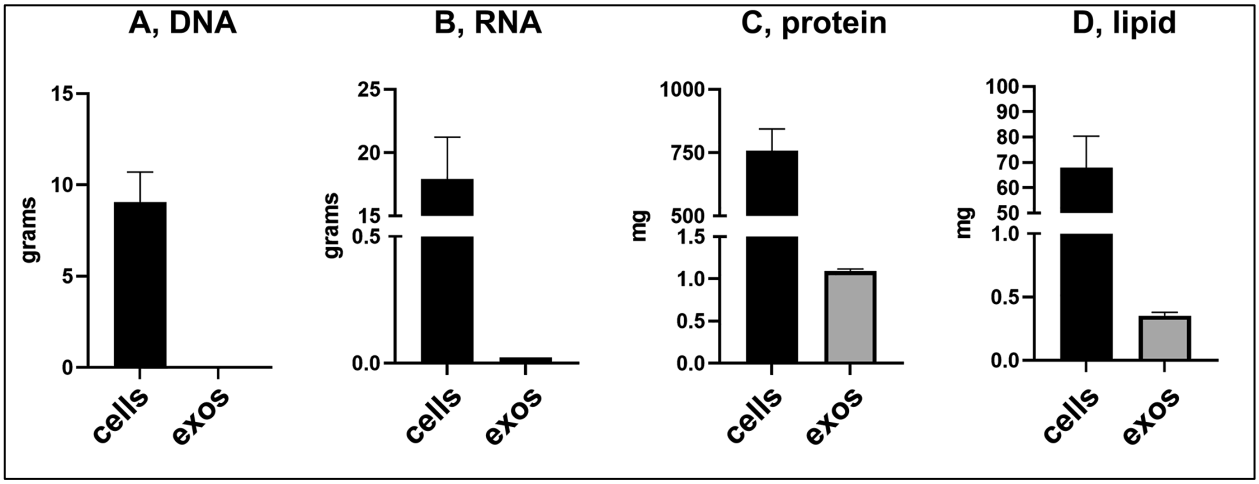

***Figure S2**. Electron microscopy reveals transformation of materials at each step of the mRNA loading process.* Transmission electron micrographs of (left panel) purified Antares2 mRNA, (center left panel) purified 293F-derived exosomes, (center panel) cationic lipid (CL) solution, (center right panel) lipid-coated mRNA solution, and (right panel) mRNA-loaded exosome formulation. Samples were examined at the concentrations used in the formulation process, were dried on coated EM grids, and stained with uranyl acetate to highlight structural properties. RNA alone showed no noticeable profile, exosomes alone displayed a donut-shaped or cup-shaped morphology due to sample drying, while the cationic lipids had the appearance of simple lipid droplets. Mixing mRNA and cationic lipid resulted in the formation of lipid-coated mRNA lattices with deeply striated, multilamellar structure (see inset in center right panel), due to the electrostatic phosphate-headgroup interactions and the compaction of these structures by the attractive force of water. Mixing these solutions with exosomes led to the disappearance of the deeply striated lipid-coated mRNA particles and the appearance of larger exosomes swollen by the uptake of the lipid-coated mRNAs.

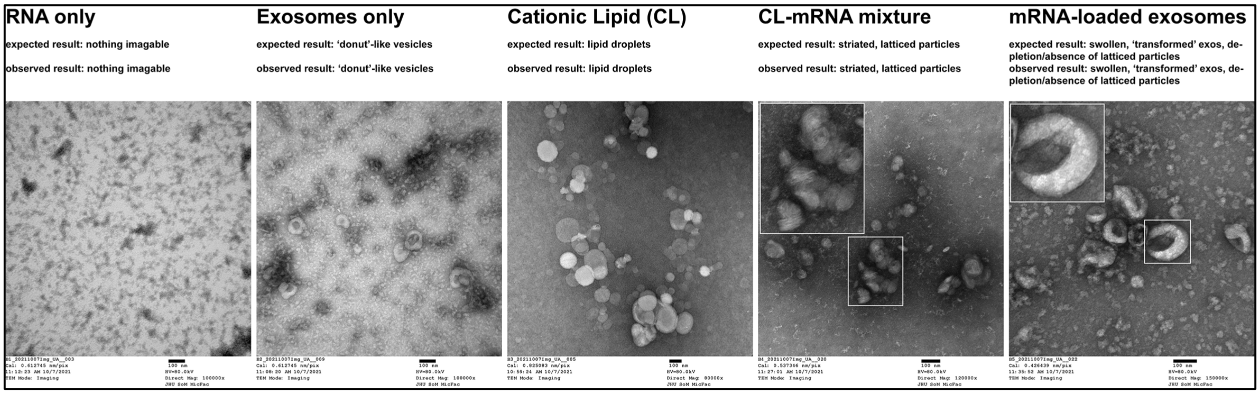

Figure S3. *Equivalent growth of vaccinated and control animals*. Body mass of all mice was measured over the course of the study and plotted as average +/- the standard error of the mean, relative to the body mass at the initiation of the trial, with groups reported as (grey lines and circles) control mice, (orange lines and squares) lower dose-treated mice, and (rust lines and triangles) higher dose-treated mice.

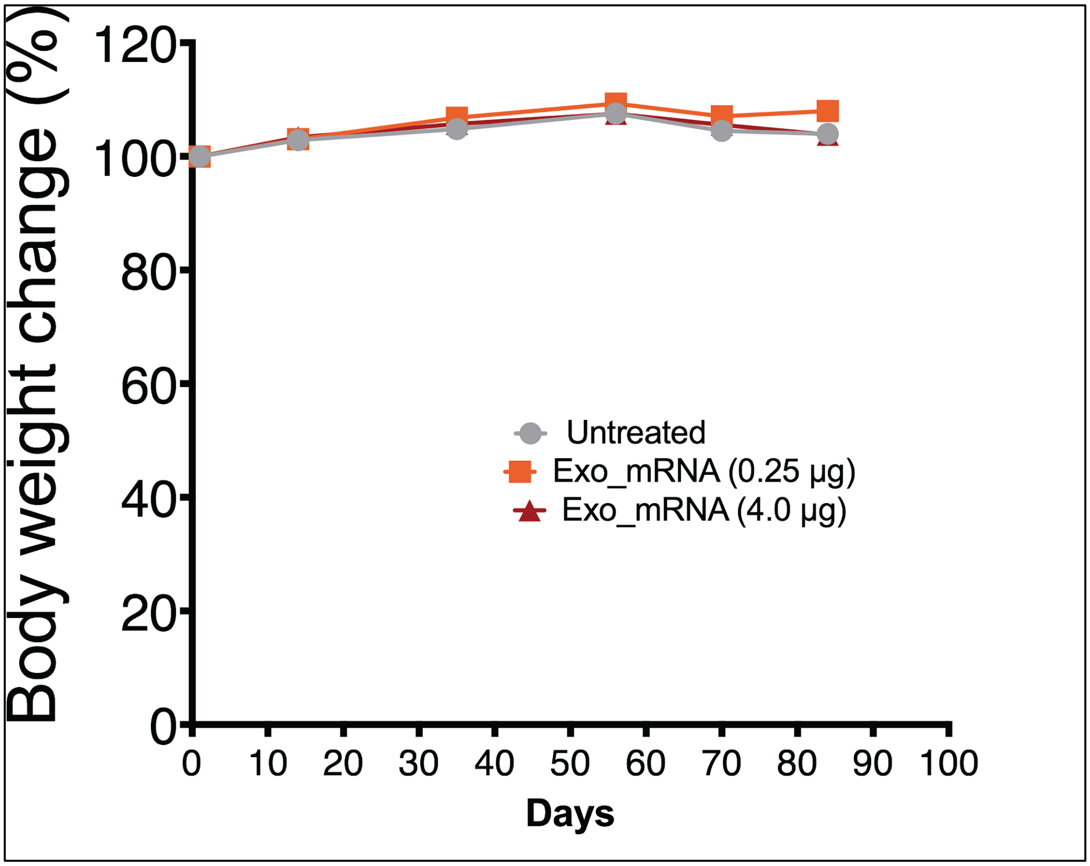

Figure S4. *Vaccination has no detectable effect on blood cell populations*. Splenocytes were interrogated by flow cytometry using antibodies specific for (A) B220, (B) Ly6C, (C) CD11c, and (D) CD3. $CD3^+$ cells were further differentiated by staining for (E) CD4 and (F) CD8. No statistically significant differences were detected in these subpopulations of white blood cells.

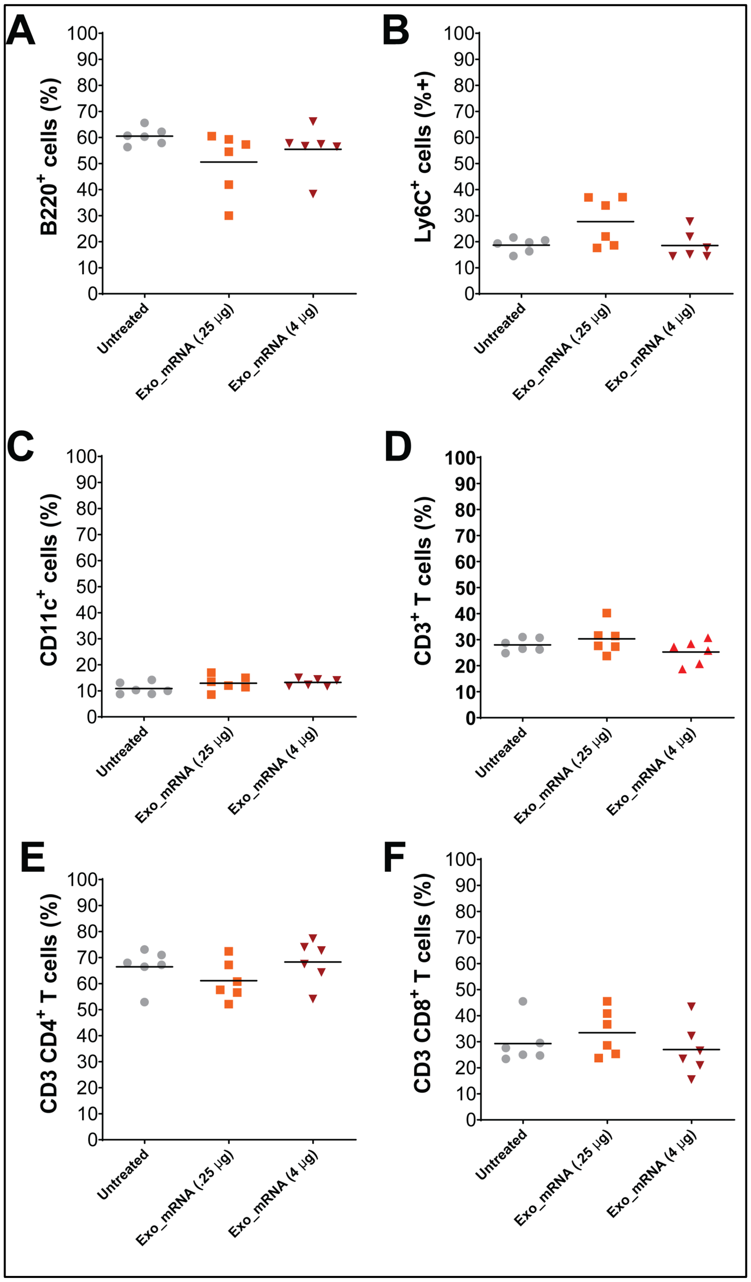

Supplement: Figures S1–S3 [file mmc1.pdf]
